# Threshold Levels of Gfi1 Maintain E2A Activity for B Cell Commitment *via* Repression of Id1

**DOI:** 10.1371/journal.pone.0160344

**Published:** 2016-07-28

**Authors:** Jennifer Fraszczak, Anne Helness, Riyan Chen, Charles Vadnais, François Robert, Cyrus Khandanpour, Tarik Möröy

**Affiliations:** 1 Institut de recherches cliniques de Montréal (IRCM), Montréal, Québec, Canada; 2 Département de microbiologie, infectiologie et immunologie, Université de Montréal, Montréal, Québec, Canada; 3 Division of Experimental Medicine, McGill University, Montreal, Canada; 4 Département de médecine, Université de Montréal, Montréal, Québec, Canada; 5 Department of Hematology, University Hospital, Essen, Germany; B.C. Cancer Agency, CANADA

## Abstract

A regulatory circuit that controls myeloid versus B lymphoid cell fate in hematopoietic progenitors has been proposed, in which a network of the transcription factors Egr1/2, Nab, Gfi1 and PU.1 forms the core element. Here we show that a direct link between Gfi1, the transcription factor E2A and its inhibitor Id1 is a critical element of this regulatory circuit. Our data suggest that a certain threshold of Gfi1 is required to gauge E2A activity by adjusting levels of Id1 in multipotent progenitors, which are the first bipotential myeloid/lymphoid-restricted progeny of hematopoietic stem cells. If Gfi1 levels are high, Id1 is repressed enabling E2A to activate a specific set of B lineage genes by binding to regulatory elements for example the *IL7 receptor* gene. If Gfi1 levels fall below a threshold, Id1 expression increases and renders E2A unable to function, which prevents hematopoietic progenitors from engaging along the B lymphoid lineage.

## Introduction

B-lymphocytes are the principal antibody producing cells and are indispensable for an efficient humoral immune response. B cell differentiation begins in the bone marrow (BM) by the generation of multipotent progenitors (MPPs) from hematopoietic stem cells (HSCs) [1, 2]. A subset of MPP cells that express Flt3 receptor, called lymphoid primed multipotent progenitors (LMPPs) [3, 4] become committed to the lymphoid lineage and generate the common lymphoid progenitors, CLPs [3, 5, 6]. Under specific conditions, both CLPs and LMPPs can generate T and B cells [7–9]. Commitment towards the B cell lineage starts after the CLP stage, when cells begin to express the marker B220 and to increase Rag1 expression. These cells, called pre-pro B cells, complete B-lineage commitment by differentiation into CD19 expressing pro B cells. They continue to differentiate along multiple stages until functional effector B cells [10].

A very complex network involving many transcription factors and cytokines such as Flt3L [11, 12] and IL-7 [13] [14] controls lymphoid commitment and B cell differentiation. B lineage specification is supported by the expression of three major transcription factors, E2A [15–17], Ikaros [18, 19] and PU.1 [20, 21] in early progenitor cells including HSCs and MPPs, allowing the formation of LMPPs. The expression levels of these three transcription factors in progenitors are known to determine cell fate decisions. For instance, the transcription factor PU.1 regulates B lymphoid versus myeloid cell lineage choice in a dose dependent manner in hematopoietic progenitors, in particular in MPPs [22–24]. Moreover, in CLPs, E2A controls the expression of EBF1, which is a major transcription factor in B cell differentiation [25, 26]. EBF1 acts in concert with E2A and Foxo1 to regulate genes essential for B cell development such as Pax5 [27]. These findings propose a transcriptional regulatory network that appear to function in a recurring manner to govern cell fate choices, especially in progenitors such as LMPPs that retain multilineage potential [5, 28].

The transcription repressor Gfi1 is a key element of this network orchestrating progenitor cell fate between myeloid and lymphoid lineages [29]. Indeed, Gfi1 deficient mice show impaired B cell differentiation, which can be partially rescued by reducing PU.1 expression levels [23], but its role in this complex transcriptional network is not fully understood.

To further understand how Gfi1 is functioning in early B lymphoid commitment and differentiation, we decided to study its participation in transcription factor regulatory circuits in early lymphoid and multipotential progenitors. We report here that a threshold level of Gfi1 protein expression is required to support B cell differentiation. We identify LMPPs as critical hematopoietic progenitors that require high levels of Gfi1 expression to repress Id1 to specifically sustain B-cell commitment. We propose a model in which Gfi1 is required to maintain Id1 at low levels, which leave E2A active to ensure the expression of those E2A target genes that are important for B cell differentiation.

## Materials and Methods

### Mice

The Institutional Review Board of the IRCM approved all animal protocols and experimental procedures were performed in compliance with the IRCM guidelines. The animals were euthanized by CO_2_ and all efforts were made to minimize suffering. GFI1 KO, GFI1 KI, GFI1-GFP and GFI1-P2A mice used in this study have been described previously [30–34]. GFI1 KD mice were generated following a previously described strategy to generate GFI1 KI mice [32, 35]. GFI1 KI mice were obtained by inserting the human GFI1-encoding cDNA into the murine Gfi1 locus (32). In the KD mice, the targeted locus still retained the neo cassette in an antisense direction leading to a low expression of the human GFI1 knock-in transgene. MB1-cre mice were obtained from Jackson laboratories (Bar Harbor, Maine, USA) [36]. Mice have been bred on C57BL/6 background for at least 10 generations and were maintained in Specific-Pathogen-Free Plus environment.

### OP9 and OP9-DL1 culture

For the B cell differentiation, sorted HSCs, MPPs, LMPPs, CLPs and pre-pro-B cells were cultured on plated OP9 cells in opti-alpha-modified Eagle Medium (OptiMEM) supplemented with IL-7 (10 ng/mL, Peprotech) and Flt3L (5 ng/mL, peprotech). After 10–12 days of co-culture, cells were analysed by flow cytometry (LSR I, BD Biosciences). For the T cell differentiation, CLPs or LMPPs were sorted and cultured on OP9 cells expressing DL1 in OptiMEM medium with SCF (10 ng/mL, R&D systems), Flt3L (5 ng/mL, Peprotech) and IL-7 (1 ng/mL, Peprotech). After 18–21 days of co culture, cells were analyzed by flow cytometry. For the myeloid differentiation, sorted LMPPs were cultured on plated OP9 cells in OptiMEM supplemented with myeloid cytokine mix described previously [37]. After 10 days, cells were analyzed by flow cytometry for Gr1 and Mac1 expression. Morpholino experiments were done with LSK cells sorted by flow cytometry were cultured in the presence of Flt3L and Il-7 for 1 hr under stroma-free conditions. Morpholinos were added to the culture in the presence of EndoPorter followed the manufacturer's instructions (Gene Tools, USA). After 4 hrs progenitor cells were transferred onto OP9 cells. After 7 days, cells were treated a second time with morpholinos and co-cultured on OP9 cells. B cell differentiation was assessed by flow cytometry for B220, CD43 and CD19. The OP9 and OP9-DL1 cells were kindly provided by Pr. Juan Carlos Zúñiga-Pflücker.

### Chromatin immuno-precipitation (ChIP) assay

Histone acetylation and E2A ChIPs were performed on 0.1 x10^6^ and 0.5 × 10^6^ sorted LSK cells, respectively, cross-linked with 0.1% or 1% formaldehyde for 8 minutes and quenched with 125mM glycine. Samples were immuno-precipitated with 2 μg anti-E2A antibody (N-649 X; Santa Cruz Biotechnology), anti-H3K9ac antibody (Ab4441; Abcam), anti-H3K27ac antibody (Ab4729; Abcam) or control anti-IgG normal rabbit antibody (12–370; Millipore) coupled with Dynabeads beads coated with Protein G and A (Life Technologies). ChIP DNA was analyzed by quantitative PCR (qPCR) using SYBR green. The enrichment (relative to Input and a negative control intergenic region) was calculated using the ΔΔCt method. Primers are in S1 Table.

### Western blot analysis

Cells were lysed in NP-40 buffer in the presence of protease inhibitor cocktail (Complete Mini, Roche Diagnostics). The following antibodies were used for immunoblotting and co-immuno-precipitation: Anti PU.1 (H-135, Santa Cruz), anti Id1 (C-20, Santa Cruz), anti Id2 (C-20, Santa Cruz), anti Id3 (C-20, Santa Cruz), anti E2A (N-649, Santa Cruz), anti Lamin B (C-20, Santa Cruz), anti Gfi1 (R&D systems) and anti β-Actin (Ac-15, Sigma-aldrich).

### Expression profiling

Expression profiling was carried out using Affymetrix arrays according to published procedures [38]. Raw data files were analyzed using the R Software ^[R reference]^, including the affy [39] and Limma packages [40]. Data was normalized using the Robust Multi-array Average (RMA) approach.

### Morpholinos

LSK cells were sorted and cultured in the presence of Flt3L and IL-7 for 1 hr under stroma-free conditions. Morpholinos were added to the culture in the presence of EndoPorter followed the manufacturer's instructions (Gene Tools, USA). After 4 hours, progenitor cells were transferred on OP9 cells. After 4 days, the cells were harvested from the co-culture and cultured in the presence of new cytokines and morpholinos with Endoporter for 4 hours. Then the cells were transferred on a new fresh OP9 cell layer. Every 4 days, cells were transferred on new OP9 cells and new cytokines were added.

The morpholino sequences: Anti-Id1 #1: CGGCACTGCCACTGGCGACCTTCAT; Anti-Id1 #2: AGAACAGAGTGTGGGAAGAGAACAA; Control: CCTCTTACCTCAGTTACAATTTATA were used as previously described in kosan *et al*. [37].

### Statistical analysis

Two tailed student’s t-test was used to calculate p-values where indicated. A p-value ≤0.05 was considered as statistically significant.

### GEO accession codes for publicly available data sets

E2A, Foxo1 and EBF1, GSE21978; Brg1 and Ikaros, GSE66978; PU.1, GSE21512.

Supplemental method about FACS stainings and RNA isolation are provided in S1 Appendix.

## Results

### The control of B cell development required a certain level of Gfi1

**Fig 1:**
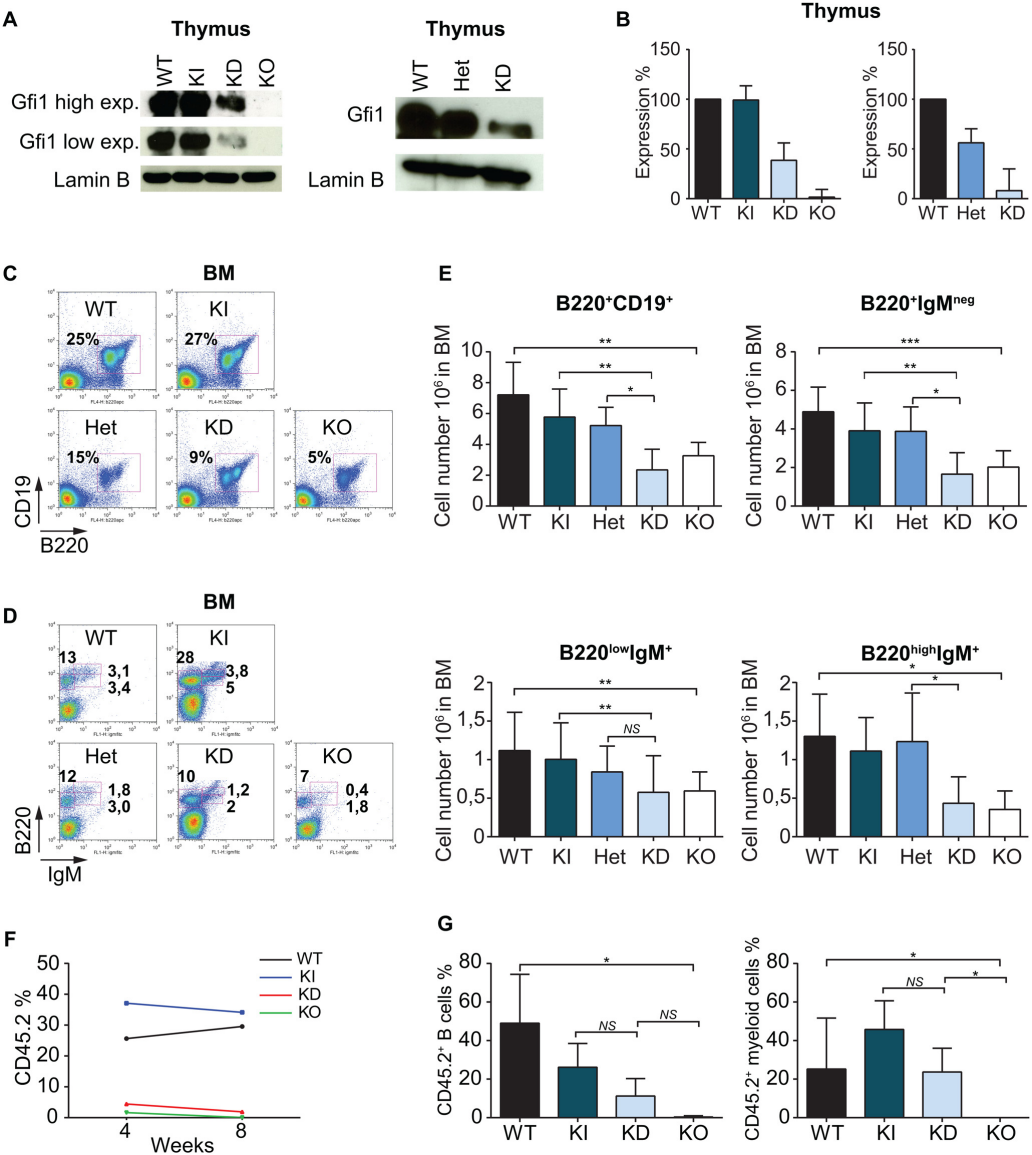
The control of B cell development by Gfi1 is dose dependent. (A) Western blot of Gfi1 levels in thymocytes isolated from Gfi1 WT, KI, Het, KD and KO mice. Lamin B was used as a loading control. Low exp. = Low exposure, high exp. = high exposure. (B) Semi quantification of relative Gfi1 expression in thymocytes from WT, KI, Het, KD and KO mice with ImageJ software by using chemiluminescent film system. (C) Flow cytometry analysis of BM B220^+^CD19^+^ B cells from WT, KI, Het, KD and KO mice. (D) Flow cytometry analysis of BM B cells from WT, KI, Het, KD and KO mice. Cells were stained for B220 and IgM surface markers. Each plot is representative of the experiment average. (E) Absolute number of BM B220^+^CD19^+^ B cells, BM B220^+^IgM^neg^, BM B220^low^IgM^+^ and BM B220^high^IgM^+^. (F) and (G) CD45.1 mice were lethally irradiated (9,5 Gy) and transplanted with an equal number of CD45.1 BM cells and CD45.2 BM cells from WT, KI, KD or KO mice. After 4 and 8 weeks, blood cells from the transplanted mice were analyzed for the expression of CD45.1 and CD45.2 markers (F) and also for B220 and CD19 or Gr1 and Mac1 (G). All FACS plots in Fig 1 are representative of at least five independent experiments and a minimum of five mice were used to determine absolute numbers of B cell subsets in BM.

We have generated mutant mice by gene targeting, in which the endogenous *Gfi1* gene is replaced by the human *Gfi1* cDNA (termed “KI” for “knockin”) [32]. During the analysis of these mice, we noticed that when the Neo cassette was not deleted, the expression level of human Gfi1 was reduced to about 20–30% of the expression level of the control WT or KI mice without Neo cassette (Fig 1A and 1B) [35]. This provided us with several mouse models expressing different Gfi1 levels (Fig 1A and 1B): WT mice expressing a normal level of murine Gfi1, KI mice expressing a normal level of human Gfi1 instead of the murine Gfi1, heterozygote mice for Gfi1 (Het) expressing around 50–60% of WT Gfi1 levels, animals expressing a reduced level of human Gfi1 (termed “KD” for “knockdown”) and KO mice that were previously described [31] and completely lack Gfi1 expression (Fig 1A and 1B). We found decreased frequencies and absolute numbers of B220^+^CD19^+^, B220^+^IgM^-^, B220^low^IgM^+^ and B220^high^IgM^+^ B cells (Fig 1C–1E) in the BM of KD mice, similar to what is seen and was previously described for Gfi1 KO mice [23, 41–43] but no significant difference between Het, KI and WT mice. This suggests that a minimal threshold level of Gfi1 expression exists below which B cell differentiation is no longer maintained. Similar decreases in B cell frequencies were also seen in the BM from Mx-Cre Gfi1^flox/flox^ mice where *Gfi1* is deleted along with the neo marker gene after pIpC injections (S1A–S1C Fig), excluding an effect of the neo marker in KD mice. In Gfi1^P2A/P2A^ mutant mice (P2A), in which Gfi1 is functionally disabled by a mutation in its N-terminal SNAG repressor impairing binding to LSD1 [30], we observed the same defect in B cell frequencies and numbers as in KD and KO mice (S1D–S1F Fig). A competitive transplantation experiment showed that Gfi1 KD and, as already described, Gfi1 KO BM cells [44] were unable to efficiently reconstitute hematopoiesis in recipient mice (Fig 1F). Among CD45.2^+^ cells in the blood of the transplanted recipient CD45.1 mice, the percentage of cells expressing B220 and CD19 was also lower in mice transplanted with KD BM cells compared to mice transplanted with WT and KI cells while the percentage of myeloid cells was not affected (Fig 1G) suggesting that the defect is likely intrinsic to the transferred cells. However, since the change in B cell reconstitution is similar to the change in overall reconstitution it cannot be excluded that the defect occurs already in the HSCs or MPPs.

### Gfi1 is important at the stage where early B cell progenitors are formed

**Fig 2:**
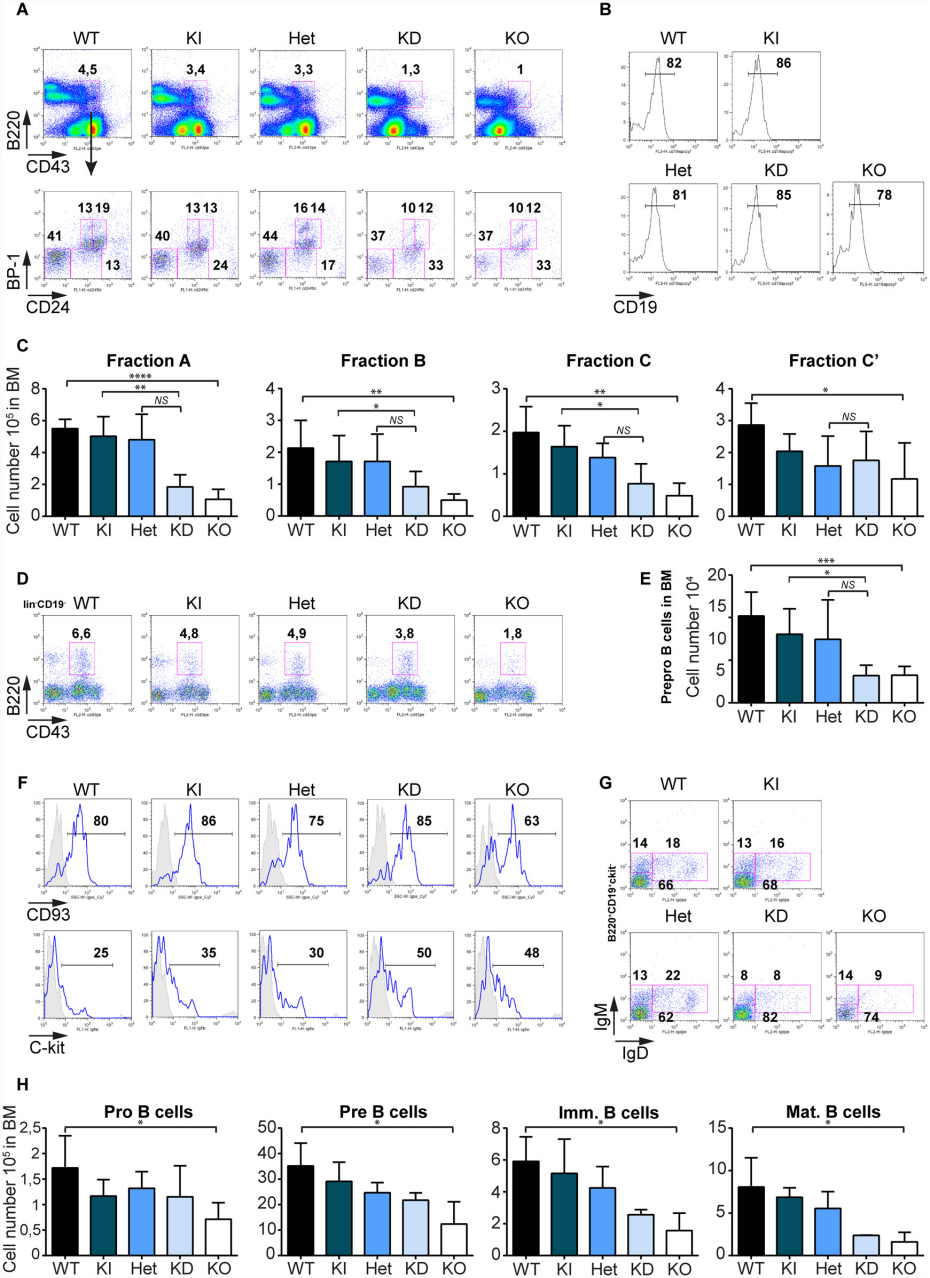
Gfi1 is important at the stage where early B cell progenitors are formed. (A) Flow cytometry analysis of B220, CD43, HSA, and BP-1 surface expression on BM cells. B220^+^CD43^+^ cells were electronically gated and analyzed for HSA and BP-1 expression to identify Fraction A (A, B220^+^CD43^+^HSA^-^BP1^−^), Fraction B (B, B220^+^CD43^+^HSA^+^BP1^−^), Fraction C (C, B220^+^CD43^+^HSA^+^BP1^+^) and Fraction C’ (C’, B220^+^CD43^+^HSA^high^BP1^+^). (B) Flow cytometry analysis of CD19 expression on pro B cells. Fraction B cells were analyzed for surface CD19 expression. (C) Absolute numbers of Fraction A-C’ from WT, KI, Het, KD and KO mice. (D) Expression of Lin^-^, CD19, B220, and CD43 on BM cells. Lin^−^CD19^−^ cells were analyzed for the expression of B220 and CD43. (E) Absolute numbers of pre-pro B cells (Lin^-^CD19^-^B220^+^CD43^+^) contained in the Fraction A from WT, KI, Het, KD and KO mice. (F) Expression of CD93 and c-Kit on pre-pro B cells from WT, KI, Het, KD and KO mice. Lin^-^CD19^-^B220^+^CD43^+^ cells were analyzed for CD93 expression or c-Kit on the surface. (G) Flow cytometry analysis of B220, CD19, c-Kit, IgM and IgD surface expression on BM cells. B220^+^CD19^+^c-Kit^-^/c-kit^+^ cells were gated for IgM and IgD expression to define Pro B cells (B220^+^CD19^+^c-Kit^+^IgM^-^IgD^-^), Pre B cells (B220^+^CD19^+^c-Kit^-^IgM^-^IgD^-^), Immature B cells (B220^+^CD19^+^c-Kit^-^IgM^+^IgD^-^) and mature B cells (B220^+^CD19^+^c-Kit^-^IgM^low/+^IgD^+^). (H) Cell numbers of Pro, Pre, Immature (Imm.) and Mature (Mat.) B cells in the BM. All FACS plots are representative of a minimum of five (A, B and D) or at least two (F and G) independent experiments. At least five mice or 2 mice were respectively used to determine absolute number of B cell fractions and B cell progenitors (G and H).

A significant reduction in the frequencies of B cell progenitors contained in both Hardy fractions A (pre-pro B cells, B220^+^CD43^+^BP-1^-^HSA^-^) and B (pro B cells, B220^+^CD43^+^BP-1^-^HSA^+^) were observed in KD and KO mice (Fig 2A) and also in P2A mice (S2A Fig). The cell surface levels of CD19 contained in fraction B were similar between all animals (Figs 2B and S2B) suggesting that the few fraction B cells remaining in KO or KD are committed B cells. Cell numbers of fractions A and B were reduced in KD, KO and P2A mice compared to WT, KI and Het (Figs 2C and S2C). Another gating strategy confirmed the reduction of the pre-pro B cell population, which is identical to Fraction A, in KD and KO mice (Figs 2D and 2E and S2D and S2E). The surface levels of CD93 and c-Kit (Fig 2F) in pre-pro B cells (lin^-^CD19^-^B220^+^CD43^+^) were similar between all the mice suggesting that the few cells present in fraction A of KO or KD mice underwent regular differentiation. Another gating strategy [45] confirmed that absence (KO) or low dose of Gfi1 (KD) lead to a decrease of B cell progenitors, whereas Het mice have a sufficient level of Gfi1 to produce normal numbers of B cell progenitors (Fig 2G and 2H).

### Gfi1 is required for the generation of LMPPs and CLPs

**Fig 3:**
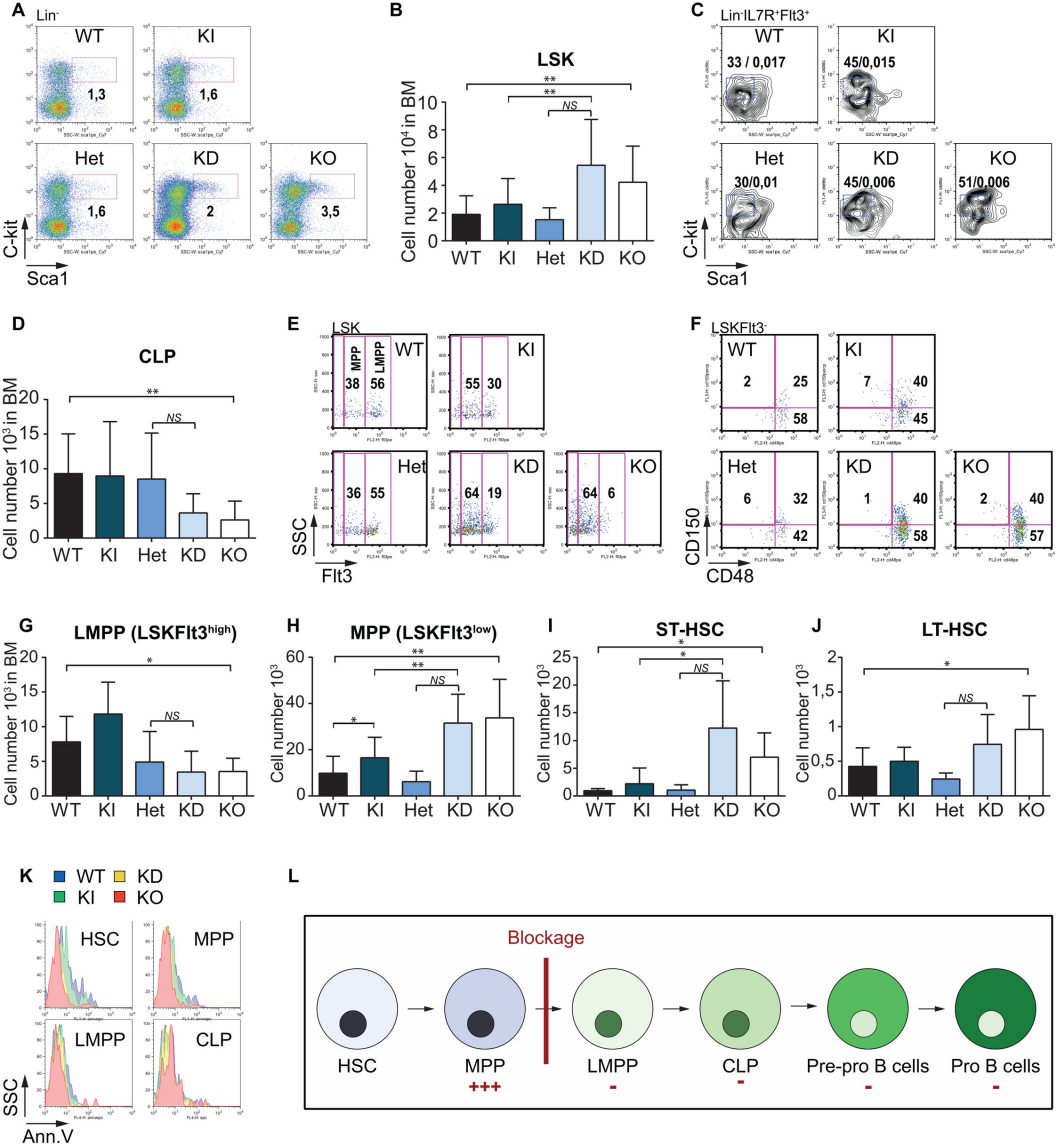
Gfi1 is required for the generation of LMPPs. (A) FACS analysis of LSK cells. BM cells from WT, KI, Het, KD and KO mice were analyzed for Lin^-^, c-Kit and Sca1 expression. (B) Absolute number of LSK cells in BM from WT, KI, Het, KD and KO mice. (C) FACS analysis of common lymphoid progenitors (CLP, Lin^-^IL7R^+^Flt3^+^Sca1^low^c-Kit^+^) in the BM of WT, KI, Het, KD and KO mice. The percentages at the left and at the right on the dot plots respectively indicate the CLP relative frequency and the CLP absolute frequency. (D) Absolute numbers of CLP in the BM of WT, KI, Het, KD and KO mice. (E) LSK cells were analyzed for Flt3 expression in the BM of WT, KI, Het, KD and KO mice. MPP (Lin^-^Sca1^+^c-Kit^-^Flt3^low^) and LMPP (Lin^-^Sca1^+^c-Kit^+^Flt3^+^) are identified on the FACS plot. (F) LSKFlt3^-^ cells were analyzed for CD48 and CD150 cell surface levels. (G) Absolute numbers of LMPP (Lin^-^Sca1^+^c-Kit^+^Flt3^+^) in the BM of WT, KI, Het, KD and KO mice. (H) Absolute numbers of MPP (Lin^-^Sca1^+^c-Kit^+^Flt3^low^) in the WT, KI, Het, KD and KO BM. (I) Absolute numbers of ST-HSC (Lin^-^Sca1^+^c-Kit^+^Flt3^-^CD150^+^CD48^high^) in the WT, KI, Het, KD and KO BM. (J) Absolute numbers of LT-HSC (Lin^-^Sca1^+^c-Kit^+^Flt3^-^CD150^+^CD48^low^) in the WT, KI, Het, KD and KO BM. (K) HSC (Lin^-^Sca1^+^c-Kit^+^Flt3^-^), MPP (Lin^-^Sca1^+^c-Kit^+^Flt3^low^), LMPP (Lin^-^Sca1^+^c-Kit^+^Flt3^+^) and CLP (Lin^-^IL7R^+^Sca1^+^c-Kit^+^) were gated and analyzed for cell death by Annexin V staining. (L) Schematic representation of lymphoid development from Hematopoietic Stem Cells (HSC) to Pro B cells with the different steps including Multipotent Primed Progenitors (MPP), Lymphoid Primed Multipotent Progenitors (LMPP), Common Lymphoid Progenitors (CLP) and Pre-pro B cells. ‘**+++**’ represents the population increased in the GFI1 KD and KO mice and ‘**-**’ represents the populations decreased in the GFI1 KD and KO mice. All FACS plots are representative of at least three independent experiments. A minimum of three mice were used to determine absolute numbers of different progenitor subsets in BM.

To determine if the reduction of B cell progenitors is mostly due to an earlier defect during B cell development, we studied the LSK (Lin^-^c-Kit^+^Sca1^+^) compartment, which includes HSCs and the early lymphoid progenitors. Higher frequencies of LSK cells were found in KO and P2A mice confirming earlier studies [44, 46] and in KD mice compared to, Het, KI and WT animals (Figs 3A and 3B and S3A, S3D). Further, frequencies and absolute numbers of CLPs (Lin^-^IL7R^+^Flt3^+^Sca1^med^c-Kit^med^) were severely reduced in KO, P2A and KD mice (Figs 3C and 3D and S3B). Although a similar effect of Gfi1 was also observed for LMPPs (Lin^-^Sca1^+^c-Kit^+^Flt3^high^), which were severely decreased in KD, KO and P2A mice (Figs 3E, 3G and S3C), both MPP (Lin^-^c-Kit^+^Sca1^+^Flt3^low^) and ST-HSC progenitors (Lin^-^c-Kit^+^Sca1^+^Flt3^-^CD48^high^CD150^+^) were equally increased in KD and KO mice compared to KI or WT mice (Fig 3F and 3H–3J) but the frequencies of these two subsets within the LSKFlt3^low^ population was similar between the five different mouse mutants (KO, KD, KI, P2A and WT). The Gfi1 expression levels in heterozygous mice did not cause an increase in the LSK population or a decrease in the LMPP subset showing that a 50–60% expression level of Gfi1 is sufficient to support B cell differentiation. The decrease of LMPPs and CLPs in KD and KO mice is not due to an increase of the cell death compared to KI and WT mice (Fig 3K). These data suggest that differentiation blocks rather than cell death are causing the reduction in the LMPP and CLP populations (Fig 3L), when Gfi1 is severely down-regulated or absent suggesting that Gfi1 may play a role at different steps of the B cell differentiation.

### Gfi1 is essential in LMPP and CLP cells for B cell lineage specification

**Fig 4:**
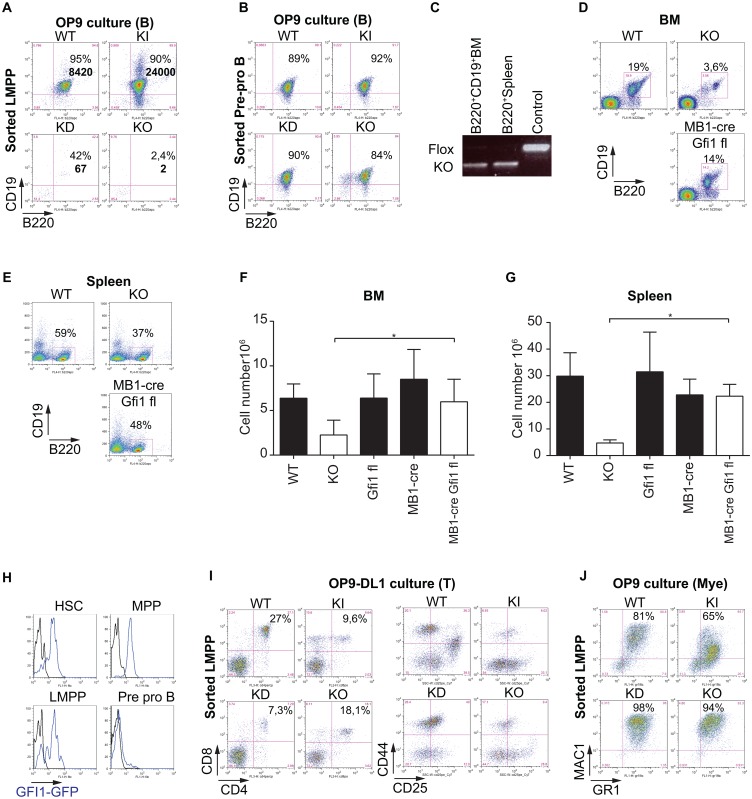
Gfi1 is essential in LMPP cells for B cell lineage specification. (A) LMPPs from WT, KI, KD and KO were sorted and cultured on stromal OP9 cells in the presence of Flt3L (5 ng/mL) and IL-7 (10 ng/mL). After 10 or 12 days of co-culture cells were analysed for B220 and CD19 expression. The cell number for each mouse is indicated in bold. (B) Pre-pro B cells were sorted and cultured on OP9 cells in the presence of Flt3L (5 ng/mL) and IL-7 (10 ng/mL). After 10 or 12 days of co-culture, cells were analyzed for B220 and CD19 expression. (C) PCR analysis showing the deletion of *Gfi1* flox allele in BM B220^+^CD19^+^ B cells and in splenic B220^+^ B cells. B220^+^CD19^+^ BM cells and B220^+^ splenic cells were sorted and lysed to extract DNA. (D) and (E) WT, Gfi1 KO (KO) and MB1-cre *Gfi1* flox/flox (MB1/Gfi1fl) mice were analyzed for B cells in the BM (D) and spleen (E). (F) Absolute numbers of B220^+^CD19^+^ B cells in WT, Gfi1 KO (KO), *Gfi1* flox/flox (Gfi1fl), MB1-cre (MB1/WT) and MB1-cre *Gfi1* flox/flox (MB1/Gfi1fl) BM. (G) Absolute numbers of B220 B cells in WT, Gfi1 KO (KO), *Gfi1* flox/flox (Gfi1fl), MB1-cre (MB1/WT) and MB1-cre *Gfi1* flox/flox (MB1/Gfi1fl) spleens. (H) BM cells from Gfi1-GFP mice were gated for HSC, MPP, LMPP, pre-pro B cell (Pre-pro B) and GFP expression (blue line) was measured in each population. (I) Sorted LMPPs from WT, KI, KD and KO mice were cultured on OP9-DL1 stromal cells in the presence of SCF (10 ng/mL), Flt3L (5 ng/mL) and IL-7 (1 ng/mL). After 15 or 21 days of co-culture, cells were analyzed for CD44, CD25, CD4 and CD8 expression. (J) Sorted LMPPs from WT, KI, KD and KO were cultured on OP9 cells in the presence of myeloid cytokine mix as previously described [37]. After 10 days, cells were analysed for Gr1 and Mac1 expression by flow cytometry. FACS plots are representative of at least three independent experiments.

HSCs, MPPs, LMPPs or CLPs from KO and KD mice were unable to direct B cell differentiation *in vitro* on OP9 cells (Figs 4A and S4A–S4C). However, sorted pre-pro B cells were able to generate B220^+^CD19^+^ cells after co-culture on OP9 cells (Fig 4B) suggesting that loss of Gfi1 affects B cell differentiation potential at the transition between MPPs and LMPPs. In addition, Gfi1 deletion after the pre-pro B cells stage (equivalent to Hardy Fraction A) using MB1-Cre Gfi1^flox/flox^ mice has almost no effect on B-cell differentiation since the frequency of B220^+^CD19^+^ cells in the BM and B220^+^ cells in the spleen were close to WT levels (Figs 4C–4G and S4D), suggesting that Gfi1 is no longer required at this stage. The fact that expression of Gfi1 drops to very low levels at the pre-pro B cell stage is in agreement with this (Fig 4H). Although LMPPs from Gfi1 KD or KO mice were unable to sustain B cell differentiation, they maintained their ability to differentiate into T and myeloid cells (Fig 4I and 4J). Similarly, CLPs from KD or KO mice could not differentiate into B cells, but reduced levels (KD) of Gfi1 were sufficient to allow CLPs to differentiate into T cells (S4E Fig).

### The B cell program is impaired in MPPs and LMPPs in Gfi1 KO and KD mice

**Fig 5:**
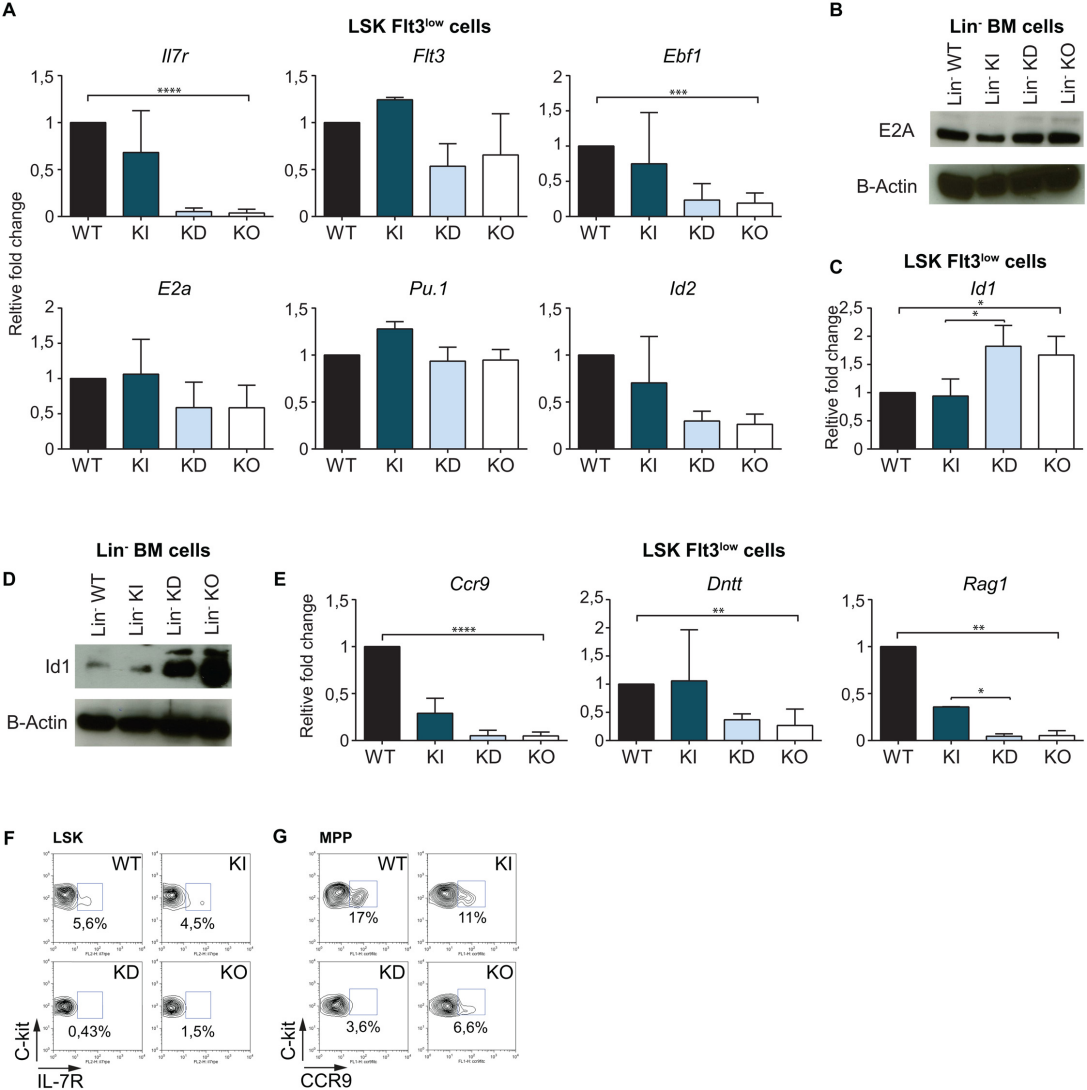
The B cell program is impaired in MPPs and LMPPs in Gfi1 KO and KD mice. (A) Analysis of *Il7r*, *Flt3*, *Ebf1*, *Pu*.*1*, *E2a* and *Id2* gene expression by RT-qPCR in sorted LSKFlt3^-/low^ cells from WT, KI, KD and KO mice. All values were normalized to the expression of the *Gapdh* gene and are presented relative to cDNA from WT mice. (B) E2A protein levels from lineage negative cells from WT, KI, KD and KO BM. β-Actin levels represent the loading control. (C) Analysis of *Id1* expression by RT-qPCR in sorted LSKFlt3^-/low^ from WT, KI, KD and KO mice. The values were normalized to the expression of *Gapdh* and are presented relative to cDNA from WT mice. (D) Id1 protein levels in lineage negative cells from WT, KI, KD and KO BM. β-Actin expression represents the loading control. (E) Analysis of E2A target gene (*Ccr9*, *Dntt* and *Rag1*) expression by RT-qPCR in sorted WT, KI, KD and KO LSKFl3^-/low^. The values were normalized to the expression of *Gapdh* and are presented relative to cDNA from WT mice. (F) IL-7R cell surface levels on WT, KI, KD and KO LSK cells. (G) CCR9 cell surface levels on WT, KI, KD and KO MPP cells. For all PCR data, at least three independent experiments were performed, each done in triplicates. FACS plots are representative of at least two (G) or three (F) independent experiments.

A comparison of mRNA expression profiles by microarray analysis of LSK cells sorted from WT and KO mice showed that the expression of genes implicated in B cell differentiation such as *Il7r*, *Flt3*, *Ebf1* were reduced in the absence of Gfi1 (S5A Fig). The reduction of *Il7r*, *Flt3*, *Ebf1* expression in KD and KO LSK cells was confirmed by real time PCR (S5B Fig). In addition, *Pu1* and *E2a* [16, 47] levels did not change with Gfi1 levels in LSK, as observed by microarray analysis and real time PCR (S5A and S5B Fig). Since the LSK subsets are different between KO, KD and control mice, we verified the expression of these genes in the LSK Flt3^low/-^ cells present in all mice, and found the same tendency, i.e. a severe or a low decrease in *Il7r*, *Flt3*, *Ebf1* expression in KD and KO mice compared to WT and KI animals and no change for *E2a* and *Pu*.*1* expression (Fig 5A). We also found no difference in PU.1 and E2A expression in Lin^-^ cells between the different mice (Figs 5B and S5C). However, the expression of *Id1*, an E2A inhibitor [48] but not *Id2* or *Id3* was significantly higher at mRNA and protein levels than controls in KO and KD mice (Figs 5C and 5D and S5A and S5B, S5D). This was in agreement with the decrease of *Il7r*, *Ebf1* [25] and *Flt3*, since they are E2A target genes. Moreover, other E2A targets that are essential for B cell commitment such as *Ccr9*, *Rag1*, *Dntt* or E2A target genes involved in lymphoid development like *Notch1* [16] were also down-regulated in KD and KO progenitor cells compared to controls (Figs 5E and S5A, S5E). In addition, compared to control mice, KD and KO mice showed a significantly lower frequency of IL-7R^+^ cells in the LSK population and CCR9^+^ cells in the MPP subset (Fig 5F and 5G). This indicated that loss (KO) or a severe down-regulation (KD) of Gfi1 leads to a higher expression of Id1 in the progenitor cell subset, and lower expression levels of genes essential for B cell lineage.

### Gfi1 regulates Id1 expression and by extension E2A targets for B cell differentiation

**Fig 6:**
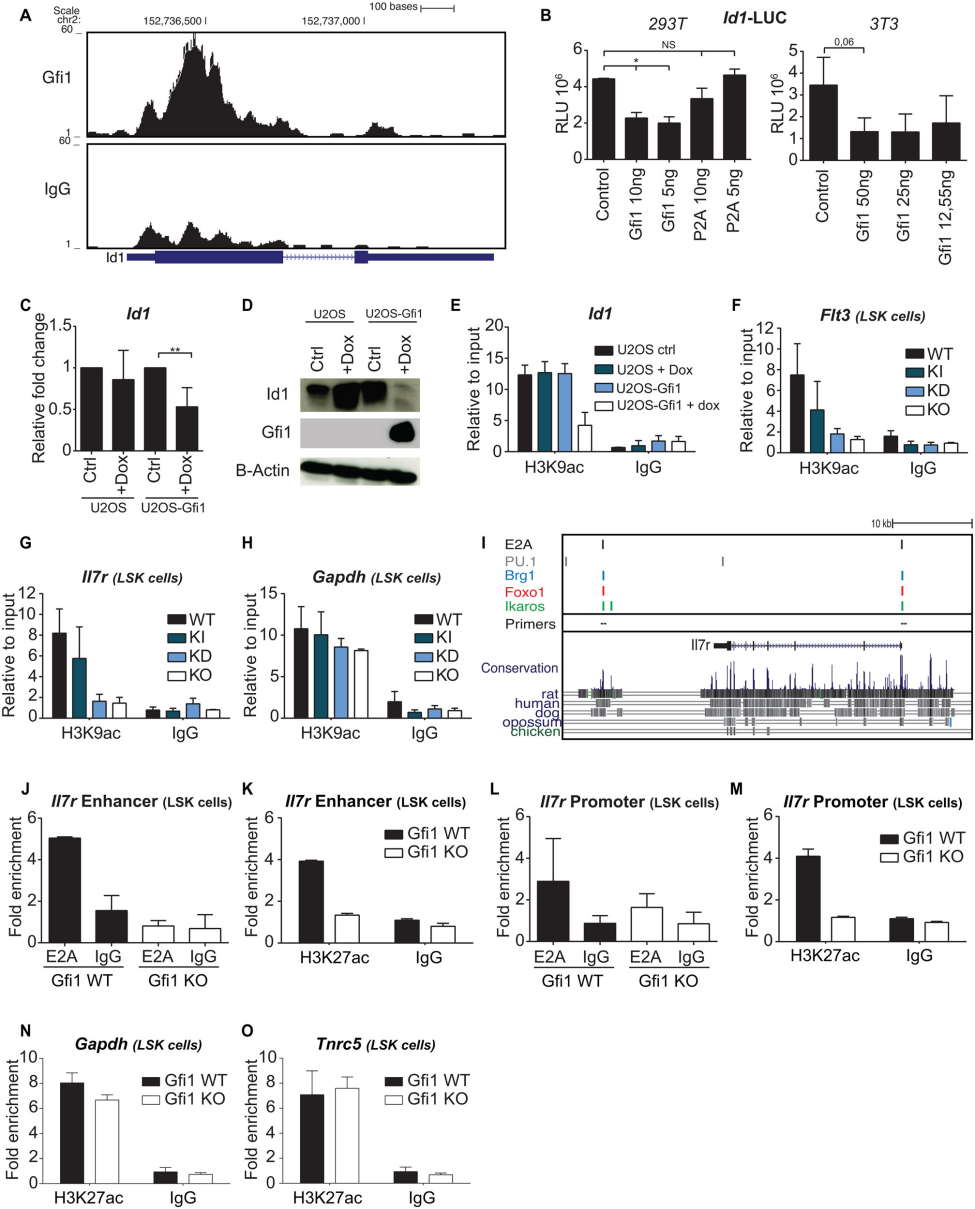
Gfi1 regulates Id1 expression and by extension E2A targets for B cell differentiation. (A) ChIP-seq data for Gfi1 and control IgG binding over the *Id1* locus in MLL-ENL transformed cells. (B) 293T cells or 3T3 cells were transfected with a luciferase reporter fused to the *Id1*promoter (Addgene, plasmid #16048) and pcDNA3.1 empty vector (EV), pcDNA3.1 with *Gfi1* (Gfi1) or pcDNA3.1 with mutant of *Gfi1* (P2A) (for 293 T cells only) in varying concentrations. Data were normalized for transfection using β-galactosidase and are presented as average relative luciferase units ± SD and are representative of two independent experiments. (C-D) U2OS and U2OS-Gfi1 (U2OS cell line in which Gfi1 can be induced with doxycycline) were treated with or without doxycycline (1 μg/mL) for 16 hours. Untreated U2OS (Ctrl), U2OS treated with doxycycline (Dox), untreated U2OS-Gfi1 (Ctrl) and U2OS-Gfi1 treated with doxycycline (Dox) cells were used for RNA (C) or protein extraction (D). Expression of *Id1* was measured and normalized to the expression of *Gapdh* and is presented as the fold change relative to cDNA from untreated U2OS cells or untreated U2OS-Gfi1 (C). β-Actin was used as a loading control (D). (E) H3K9ac ChIP-qPCR at the *Id1* promoter in U2OS and U2OS-Gfi1 cells treated with or without doxocycline (1 μg/mL) for 16 hours. (F-H) H3K9ac enrichment by ChIP-qPCR at the promoters of *Flt3*, *Il7r* and *Gapdh* from LSK cells sorted from WT, KI, KD and KO mice. (I) E2A, PU.1, Brg1, Foxo1 and Ikaros targeted regulatory regions from RAG1 null pro-B cells by ChIP-seq across the *Il7r* locus using published data [27]. The localization of primers used for ChIP-qPCR in Figs 6 G-M is represented in the Figure. (J) E2A ChIP enrichment at an *Il7r* 3’ putative enhancer in Gfi1 WT and KO sorted LSK cells. (K) H3K27ac enrichment by ChIP-qPCR at the *Il7r* 3’ putative enhancer in Gfi1 WT and KO sorted LSK cells. (L) E2A ChIP enrichment at an *Il7r* promoter in Gfi1 WT and KO sorted LSK cells. (M) H3K27ac ChIP enrichment at an *Il7r* promoter in Gfi1 WT and KO sorted LSK cells. (N) and (O) H3K27ac ChIP enrichment at *Gapdh* and *Tnf* regions in Gfi1 WT and KO sorted LSK cells. For the ChIP experiments, error bars represent standard deviation of three independent experiments for Figs 6 E-H and two independent experiments for Figs 6 J-N.

Gfi1 occupies the *Id1* promoter in MLL-ENL transformed cells suggesting a direct regulation of Id1 expression by Gfi1 (Fig 6A) [49, 50]. A reporter gene assay using an *Id1* promoter-driven luciferase gene showed that Gfi1, but not the non-functional Gfi1P2A mutant repressed Id1 expression in both 293T cells and 3T3 cells (Fig 6B). *Id1* mRNA levels were not affected by loss or low doses of Gfi1 in pre-pro B cells where Gfi1 expression is absent (S6A Fig) and *Flt3* and *Il7r* mRNA are readily detected. In U2OS cells, in which Gfi1 can be induced by doxycycline, *Id1* mRNA expression was reduced upon induction of Gfi1 as well as its protein level (Fig 6C and 6D). This reduction of Id1 levels correlated with a decreased acetylation of histone H3 at lysine 9 (H3K9ac), a marker of transcriptionally active genes, at the *Id1* promoter (Fig 6E), suggesting a transcriptional repression of the *Id1* gene by Gfi1, a known recruiter of histone deacetylases [51]. Moreover, the higher expression of Id1 in Gfi1 KD and KO mice correlated with a decrease in H3K9ac at the E2A target genes *Flt3* and *Il7r*, but not at the control *Gapdh* promoter (Fig 6F–6H). Interestingly, we observed that E2A binds to an *Il7r* evolutionary conserved putative enhancer region as well as to the *IL7r* promoter in LSK cells (Fig 6J and 6L), using previously published E2A ChIP-seq data from murine pro-B cells (Fig 6I; [27]). Brg1, Foxo1 and Ikaros were also found to occupy the same sites as E2A at the IL7r locus (Fig 6I) and often target the same regions as E2A at other B-cell specific genes that are affected by Gfi1 (S6B–S6H Fig). Upon the loss of Gfi1, however, binding of E2A was no longer detected at the *Il7r* enhancer site in sorted LSK cells by ChIP-qPCR (Fig 6J) and also coincided with a reduction in H3K27 acetylation (H3K27ac) enrichment, a marker for transcriptionally active promoters and particularly for active enhancers, at this region (Fig 6K) [52]. Furthermore, E2A binding to the *Il7r* promoter was also decreased in the absence of Gfi1 (Fig 6L) and a reduced enrichment of H3K27ac at *Il7r* promoter was also found (Fig 6M), which was not the case at the control promoter regions of *Gapdh* or *Tnrc5* (Fig 6N and 6O). These data suggest that the reduction of Gfi1 expression beyond a certain threshold leads to increased levels of Id1 expression, which in turn inhibits E2A and prevents it from activating B-cell specific genes.

### Reduced expression of Id1 in LSK cells rescues B cell differentiation

**Fig 7:**
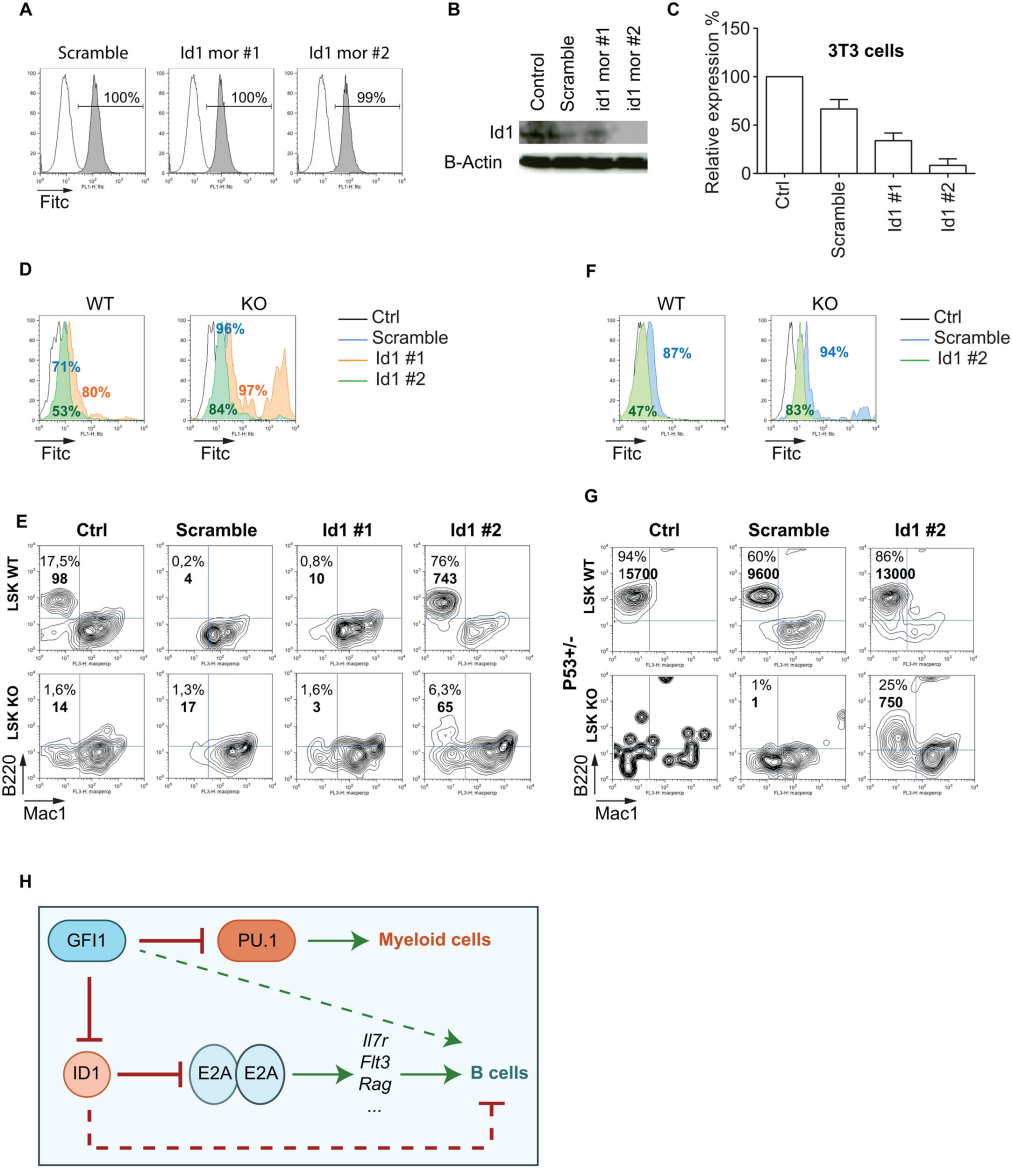
Reduced expression of Id1 in LSK cells rescues B cell differentiation. (A) 3T3 cells were transfected with control scrambled-Fitc or two different morpholinos (Fitc) (Id1 mor #1 and Id1 mor #2) targeting *Id1* following the company’s instructions. The efficiency of the transfection was measured by flow cytometry. (B) Id1 expression was measured by Western blot in 3T3 cells transfected with scrambled-Fitc, Id1 morpholino # 1 (Id1 mor #1) or Id1 morpholino # 2 (Id1 mor#2). β-Actin was used as a loading control. (C) Relative protein quantity of Id1 was measured with Image J software. (D) LSK cells from WT or KO mice were sorted and transfected or not with the control morpholino or with morpholinos directed against Id1 and cultured on OP9 cells in the presence of IL-7 and Flt3L. For each condition, the percentages of Fitc^+^ cells are indicated on the histogramme plots. Black is for the control cells, blue is for the cells transfected with the scramble, orange is for the cells transfected with the morpholino1 against Id1 (Id1 mor #1) and green is for the cells transfected with the morpholino 2 against Id1 (Id1 mor #2). (E) FACS analysis of LSK cells from WT and KO mice, transfected or not with scramble-Fitc, Id1 mor #1 or Id1 mor #2 and cultured on OP9 cells in the presence of IL-7 and Flt3L. B220, and Mac1 expression was measured after gating on the Fitc positive cells for the LSK cells transfected with morpholinos. The cell number for each condition is indicated in bold. (F) LSK cells from WT p53+/- or KO p53+/- mice were sorted and transfected or not with the control morpholino or with the morpholino directed against Id1 and cultured on OP9 cells in the presence of IL-7 and Flt3L. For each condition, the percentages of Fitc^+^ cells are indicated on the histogramme plots. Black is for the control cells, blue is for the cells transfected with the scramble and green is for the cells transfected with the morpholino 2 against Id1 (Id1 mor #2). (G) FACS analysis of LSK cells from WT, p53+/- and KO, p53+/- mice, transfected or not with scrambled-Fitc, or Id1 mor #2 and cultured on OP9 cells in the presence of IL-7 and Flt3L. B220, and Mac1 expression was measured after gating on the Fitc positive cells for the LSK cells transfected with morpholinos. The cell number for each condition is indicated in bold. (H) Schematic representation of the role of Gfi1 in B cell differentiation. It has been shown that Gfi1 inhibits PU.1 activity preventing its support of myeloid differentiation [23]. Our data suggest that Gfi1 exerts an additional function in B cell lineage commitment by inhibiting Id1 and thus allowing E2A to support the expression of targets genes such as *Il7r*, *Flt3*, *Rag* and others necessary for B cell differentiation. Green line: pathway positively regulated, Red line: pathway negatively regulated, Dashed green line: positively supports B-lineage differentiation, Dashed red line: suppresses B-lineage differentiation.

To further test whether Gfi1 regulates the Id1/E2A axis in B-lineage development, we set out to inhibit Id1 in progenitors that lack Gfi1 to rescue B cell differentiation. We used morpholinos directed against *Id1*, which were able to enter 3T3 cells at high efficiency and led to a specific reduction of Id1 expression (Fig 7A–7C). We transfected LSK cells from WT or KO mice with the control morpholino and the two specific morpholinos (Id1 mor#1 and Id1 mor#2) (Fig 7D) and followed B cell differentiation on OP9 cells. WT cells transfected with morpholino Id1 #2 differentiated well into B220^+^ cells compared to the control cells or cells transfected with control morpholino or Id1 #1 (Fig 7E). After 15 days of co-culture, few LSK cells from Gfi1 KO mice transfected with Id1 #2 were able to differentiate into B220^+^ B cells (Fig 7E). The low numbers of B220^+^ cells obtained at the end of the differentiation for both WT and KO LSK cells, make the conclusion about Id1 silencing in LSK cells from KO mice difficult. We observed that most of the WT or KO LSKs did not support the transfection with morpholinos and died. To counteract this, we used LSK cells from Gfi1KO p53^-/+^ mice and from p53^-/+^ animals as controls. We found that Gfi1KO p53^-/+^ LSKs were able to generate cells expressing B220 after transfection with Id1 #2 compared to untreated cells or cells treated with the scrambled control morpholino (Fig 7F and 7G). LSKs from p53^-/+^ animals were used as controls and developed B220^+^ cells when untreated and also when treated with the scrambled control morpholino (Fig 7F and 7G). Even though these results were obtained with cells missing one allele of p53 to counteract cell death, they indicated that reduction of Id1 expression could overcome the block in early B-cell differentiation induced by loss of Gfi1. The data suggest a model, in which besides its effect on PU.1, Gfi1 by repressing Id1 positively regulates E2A activity and E2A target genes required for B cell commitment (Fig 7H).

## Discussion

We present evidence that a threshold level of Gfi1 is required to direct B cell commitment in multipotent hematopoietic progenitors. We demonstrate that Gfi1 mediates this function by enabling the transcription factor E2A to activate a specific set of target genes that are critical for early B cell development. Loss of or reduced Gfi1 expression leads to de-repression of Id1, inactivation of E2A and ultimately the loss of expression of B cell differentiation genes. Our findings provide new insights that define Gfi1 as an essential control element of the E2A-Id1 axis.

### LMPPs and CLPs require Gfi1 threshold levels for B-lineage priming

To maintain B cell development in mice, and more specifically to maintain LMPP or production with B lineage potential, a minimal expression level of Gfi1 is required. Whereas a reduction of Gfi1 expression to 50–60% is tolerable for directing B-lineage commitment and B-cell differentiation, a reduction to 20–30%, affects early B-cell differentiation severely. This is supported by our data from experiments using mice expressing different defined levels of Gfi1 and the determination of progenitor cell numbers by flow cytometry and in vitro B-cell differentiation on OP9 cells. Our data also indicate that Gfi1 expression levels are critical at the transition from MPPs to LMPPs, since LMPP but not MPP numbers drop significantly when Gfi1 expression is below 50–60%. Gfi1 is also essential for the transition between the CLP and pre-pro B cell stage because we observed that KD and KO CLP cells were still unable to produce B cells while the KD and KO pre-pro B cells produced B cells under OP9 supported conditions. Although this suggests that expression of Gfi1 is important at different stages of B cell development, we decided to focus on the MPP/LMPP transition. Also, LMPPs, which have both lymphoid and myeloid potential [3, 53], require Gfi1 expression levels to be at least at 50–60% to sustain B-cell differentiation *in vivo* or *in vitro*. Our data also show that LMPP do not require Gfi1 at all to direct myeloid differentiation and only require a relatively low Gfi1 expression level at around 20–30% of WT levels to initiate T-cell differentiation. Why LMPPs do not need Gfi1 for T cell differentiation remains unclear. It is possible that LMPPs retain the capacity to activate pathways that can compensate for Gfi1 deficiency in T cell development. The presence of the chemokine CCR9 on the surface of LMPPs could offer an explanation, since CCR9 expressing progenitors from the BM have been shown to go directly to the thymus, bypassing the CLP and ETP stages, and differentiate into T cells [53].

### Gfi1 gauges E2A activity in LMPPs by regulating the expression of Id1

Although it is known that LMPPs have lymphoid and myeloid potential [3, 53], the mechanisms that engage these cells along a specific lineage remain incompletely understood. Transcription factors like PU.1 [21], Ikaros [18, 19], E2A [17, 47], EBF1 [25], Pax5 [54] and Foxo1 [45, 55, 56] are important in this process and several studies have linked these factors in regulatory circuits based on mathematical models [57]. The B lymphoid priming of LMPPs involves the activation of a specific set of genes including *Rag1*, *Dntt*, *Notch1*, *Ccr9*, *Il7r* and *Flt3*, all of which are targets or downstream effectors of E2A [16, 47]. Furthermore, B cells do not develop in E2A deficient mice, but T cells can be found in these animals [17, 58]. Interestingly, the regions bound by E2A at loci such as *Flt3*, *Notch1* and *Dntt* are also frequently occupied at the same position by Brg1, EBF1, Foxo1 and/or Ikaros emphasizing these areas as key regulatory regions. Our findings that Gfi1 deficient LSKs show decrease of E2A target genes and that Gfi1 deficient LMPPs lose B cell potential but retain T cell and myeloid potential similar to E2A deficient cells are in agreement with the notion that Gfi1 regulates E2A activity in hematopoietic progenitors. Our data further suggest that Gfi1 exerts this regulatory role of E2A in LSL Flt3^-^ cells by controlling the expression of Id1 [48], a protein already described to play a role in B cell fate determination [59, 60]. The first indication that Gfi1 can regulate the expression of *Id* genes came from studies with Gfi1 deficient thymocytes [43]. More recently, it has been shown that Gfi1 can directly repress *Id2* and *Id3* genes in hematopoietic progenitors, while activating the expression of *Id1* [41]. These observations regarding Id proteins stand in contrast to the findings presented in this study, which shows an increase of Id1 expression when Gfi1 expression is reduced or absent in LSK Flt3^-^ cells. However, the same team published a recent paper where they found a higher Id1 expression in the total BM from Gfi1 KO mice, comforting the data found in our study [61]. Nevertheless, we didn’t find the same observation for Id2 or Id3 expression in Gfi1 KO progenitor cells [41, 61], which could be explained by a different regulation of Id proteins during B cell development. Our data from experiments such as Ch-IP and reporter gene assays strongly suggest that Gfi1 occupies the *Id1* promoter and represses Id1 expression in LSK Flt3^-^ progenitors. Future experiments have to be done to confirm these data in primary cells especially in B cell progenitors with a ChIP-PCR for Gfi1 but technical limitations such as cell number, antibody quality have to be overcome to perform this experiment. Finally, the fact that we can measure reduced histone H3K9 lysine acetylation at the *Id1* promoter upon induction of Gfi1 expression is in agreement with this, given that Gfi1 can associate with HDACs at promoter sites [51]. Further evidence for the validity of this model comes from expression analyses, which allowed us to identify several E2A targets involved in B cell fate determination [16] that are decreased in the LSK compartment of Gfi1 KD and KO mice such as *Il7r*, *Ebf1*, *Flt3*, *Ccr9*, *Rag1*, *Dntt*, and *Notch1*. Our Id1 knock-down experiments that rescue the ability to drive B cell development in Gfi1 deficient progenitors, which we discovered express high levels of Id1, lends further support to this model. In this experiment, only severe reduction of Id1 protein levels was efficient to restore partially B cell development in Gfi1 deficient progenitors, which offers an explanation to why a previous crossing between Gfi1 KO mice and Id1 heterozygous mice was not sufficient to rescue B cell development [61]. Since Id1 deficient mice are causing a number of other defects [60, 62], experiments with morpholinos were conducted rather than with mice in which Gfi1 and Id1 were ablated at the same time. Although, we obtained only a partial rescue by knocking down Id1 in a p53^+/-^ background, which is likely due to technical limitations of the experiment owing to the fragility of Gfi1 deficient cells, the result are in agreement with our hypothesis. As previously shown, knocking down Pu.1 had a similar effect and partially rescued B-cell development in Gfi1 KO mice [23]. It is therefore conceivable that a knock down of both Pu.1 and Id1 at the same time may completely rescue B cell development in Gfi1 KO mice. It remains to be shown whether a similar increase of Id1 exists at the CLP stage but in accordance with the high expression of Id1 observed in lineage negative cells, we hypothesize that Id1 is also involved in the developmental block at this stage. However other targets could be also take part in this developmental block, but further experiments have to be performed to address this question.

### Evidence that E2A acts on the promoter of the Il7r gene via a distal enhancer element

*Il7r* is the target gene that is most affected by the absence of Gfi1 in progenitors and Gfi1 has previously been implicated in the regulation of *Il7r* in thymocytes [63]. Our findings reported here suggest that Gfi1 regulates *Il7r* expression by controlling the activity of E2A *via* Id1. Two E2A binding sites at the *Il7r* locus were identified in murine pro B cells [27]. The conservation of the sequences suggested the existence of similar E2A dependent regulatory regions at the promoter of the *Il7r* gene and at a putative 3’ enhancer region downstream of the *Il7r* locus in other species. Both elements are also bound by other regulatory transcription factors such as Brg1, Foxo1 and Ikaros, further highlighting their importance as regulatory regions. E2A and some of these factors also occupy other genes driving B cell development such as *Rag1/2*, *Dntt*, *Notch1*, *Ccr9*, *Il7r* and *Flt3*, all of which are targets or downstream effectors of E2A which are down-regulated in Gfi1 deficient cells, supporting the notion that Gfi1 regulates this particular gene set *via* the Id1/E2A axis. Using ChIP-qPCR, E2A, H3K9ac and H3K27ac were detected at both sites in Gfi1 WT progenitors, which is consistent with transcriptional activation by E2A. Strikingly, the enrichment of both E2A and H3K27ac, a histone mark that can distinguish active enhancers from inactive/poised enhancers [52], is lost or reduced in Gfi1 KO cells at the promoter and at the putative distal enhancer region. This also supports our hypothesis that threshold levels of Gfi1 are required to permit E2A to bind to and transcriptionally activate its target genes. Gfi1 exerts this function by blocking *Id1* transcription, enabling hematopoietic progenitors to maintain their potential for the B lineage.

## Supporting Information

S1 Appendix Supplemental method. (PDF)

S1 Fig The control of B cell development by Gfi1 is dose dependent. (A) Western blot Gfi1 from thymocytes isolated from Mx-cre Wt, *Gfi1* flox/flox mouse (WT) treated with pIpC and Mx-cre Tg, *Gfi1* flox/flox mice (MxcreTg) treated with pIpC to delete Gfi1. Lamin B was used as a loading control. (B) and (C) FACS analysis of bone marrow B cells from Mx-cre Wt, *Gfi1* flox/flox mouse (WT) treated with pIpC and Mx-cre Tg, *Gfi1* flox/flox mice (MxcreTg) treated with pIpC to delete Gfi1. Bone marrow cells were stained for B220 and CD19 (B) or B220 and IgM (C). (D) FACS analysis of B220 and CD19 in the bone marrow of WT and P2A mice. (E) FACS analysis of B220 and IgM in the bone marrow of WT and P2A mice. (F) Cell numbers of B220^+^ CD19^+^ cells in the WT and P2A bone marrow. At least three mice were used to determine absolute numbers of B cell subsets in bone marrow.
(PDF)

S2 Fig Gfi1 is important at the stage where early B cell progenitors are formed. (A) Flow cytometry analysis of B220, CD43, HSA, and BP-1 surface expression on bone marrow cells from WT or P2A mice. B220^+^CD43^+^ cells were electronically gated and analyzed for HSA and BP-1 expression to identify Fraction A (Fr.A, B220^+^CD43^+^HSA^-^BP1^−^), Fraction B (Fr.B, B220^+^CD43^+^HSA^+^BP1^−^), Fraction C (Fr.C, B220^+^CD43^+^HSA^+^BP1^+^) and Fraction C’ (Fr.C’, B220^+^CD43^+^HSA^high^BP1^+^). (B) CD19 expression on pro B cells from WT and P2A mice. (C) Absolute numbers of Fraction A-C’ from WT and P2A mice. (D) Expression of Lin^-^, CD19, B220, and CD43 on bone marrow cells from WT and P2A mice. Lin^−^CD19^−^ cells were analyzed for the expression of B220 and CD43. (E) Absolute numbers of pre pro B cells in WT and P2A bone marrow. At least three mice were used to determine absolute numbers of B cell subsets in bone marrow.
(PDF)

S3 Fig Gfi1 is required for the generation of LMPPs. (A) LSK cell numbers in WT and P2A bone marrow. (B) CLP numbers in WT and P2A bone marrow. (C) Cell numbers of HSC, MPP and LMPP cells in the WT and P2A bone marrow. (D) BM cells from WT, KI, Het, KD and KO mice were stained with control isotypes to determine the CLP population gate. At least three mice were used to determine absolute numbers of B cell subsets in bone marrow.
(PDF)

S4 Fig Gfi1 is essential in LMPP cells for B cell lineage specification. (A) HSC (Lin^-^Sca1^+^c-Kit^+^Flt3^-^), (B) MPP (Lin^-^Sca1^+^c-Kit^+^Flt3^low^) or (C) CLP (Lin^-^Il7R^+^Sca1^+^c-Kit^+^) were sorted and cultured on OP9 stromal cells in the presence of IL-7 (10 ng/mL) and Flt33L (5 ng/mL) for 10 or 12 days. Then cells were analyzed for B220 and CD19 expression by flow cytometry. The cell number on the FACS plots for each mouse is indicated in bold. (D) *Gfi1* flox/flox (Gfi1fl), MB1-cre (MB1/WT) mice were analyzed for B cells in the bone marrow and spleen. (E) Sorted CLPs were cultured on OP9-DL1 cells in the presence of SCF (10 ng/mL), FLT3L (5 ng/mL) and IL-7 (1 ng/mL) for at least 15 days. The cells were analyzed for CD44, CD25, CD4 and CD8 expression. All FACS plots are representative of at least two independent experiments and at least three mice were used to determine absolute numbers of B cell subsets in bone marrow and spleen.
(PDF)

S5 Fig The B cell program is impaired in MPPs and LMPPs in Gfi1 KO and KD mice. (A) Table showing the expression log2 fold change of selected up- and down-regulated genes between sorted WT and KO LSKs. (B) *Il7r*, *Flt3*, *Ebf1*, *Pu*.*1*, *E2a*, *Id1*, *Id2* and *Id3* expression in sorted LSK cells from WT, KI, KD and KO mice was measured by real time qPCR. Expression of the genes was normalized to *Gapdh* and is presented relative to cDNA from WT cells. (C) PU.1 expression in Lin^-^ cells from WT, KI, KD and KO BM was measured by western blot. β-actin was used as a loading control. (D) Id2 and Id3 expression in lineage negative cells from WT, KI, KD and KO mice measured by western blot. β-actin was used as a loading control. (E) *Ccr9*, *Notch1*, *Rag1* and *Dntt* expression in sorted LSK cells from WT, KI, KD and KO mice was measured by real time qPCR. Expression of the genes was normalized to *Gapdh* and is presented relative to cDNA from WT cells. Real time PCR are representative of at least 3 independent experiments and the western blot scans are representative of two different experiments.
(PDF)

S6 Fig Gfi1 regulates Id1 expression and by extension E2A targets for B cell differentiation. (A) *Il7r*, *Flt3* and *Id1* expression in sorted pre-pro B cells from WT, KI, KD and KO was measured by real time qPCR. Expression of *Il7r*, *Flt3* and *Id1* was normalized to *Gapdh* and presented as the fold increase relative to cDNA from WT cells. (B-H) E2A, PU.1, Brg1, EBF1, Foxo1 and Ikaros targeted regulatory regions from RAG1 null pro-B cells by ChIP-seq across the *Il7r*, (C) *Flt3*, (D) *Ccr9*, (E) *Ebf1*, (F) *Notch1*, (G) *Dntt*, (H) *Rag1* loci.
(PDF)

S1 Table Primers for mouse and human ChIP-qPCR. (PDF)

S2 Table Primers for real Time PCR. (PDF)
