# Supplementary material for: Threshold Levels of Gfi1 Maintain E2A Activity for B Cell Commitment via Repression of Id1
Source: PLoS One. 2016 Jul 28;11(7):e0160344. doi: 10.1371/journal.pone.0160344 (PMC4965025; doi:10.1371/journal.pone.0160344)
Supplement: S1 Appendix — (PDF) [file pone.0160344.s001.pdf]

Supplemental method

Flow cytometry, cell surface staining and cell sorting

The following antibodies were purchased from BD Biosciences: B220 APC (RA3-6B2), CD19 APC-Cy7 (1D3), IgM Fitc (R6-60.2), IgD PE (11-26c.2a), CD43 PE (S7), c-Kit APC (2B8), c-Kit Fitc (2B8), Sca1 Pe-cy7 (D7), Flt3 PE (A2F10.1), Flt3 APC (A2F10.1), IL7R PE (A7R34), and CD48 PE (HM48-1). CD150 Percp-cy5.5 (TC15-12F12.2) was from Biolegend, biotinylated BP-1 (6C3), biotinylated Flt3 (A2F10) and CD24 Fitc (30-F1) were from eBiosciences and CCR9 Fitc (FAB2160F) was from R&D systems. The lineage cocktail used for the LSK or CLP staining was composed by biotinylated antibodies against CD3, CD8, B220, Gr1, Mac1, Ter119, CD5 (53-7.3), IgM (R6-60.2), NK1.1 (PK136) and CD4 (RM4-5). The lineage cocktail used for pre-pro-B cell staining has been previously described (Kosan et al., 2010). Antibody incubation was performed at 4°C for 15 min in PBS, then cells were washed with PBS and analyzed on FACS LSR I (BD Biosciences). Cell sorting was performed by using a MoFlo cell sorter (cytomation) or Facs Aria III cell sorter (BD Biosciences). AnnexinV staining was performed using the AnnexinV-APC antibody and AnnexinV detection kit I (BD biosciences) by following the manufacturer's instructions. Cells were analyzed with LSR I (BD Biosciences).

RNA isolation and real-time PCR

For RNA isolation, cells were lysed in Trizol (Life technology) or FACS-sorted directly into RLT buffer with 10% of β-mercaptoethanol (RNeasy Micro kit, Qiagen). RT-PCR was performed using Superscript II (Invitrogen). Real time PCR was performed in triplicates on the ViiA7 Real time PCR machine (Life technologies) in SYBR Green Master mix (Applied Biosystems). The

expression of the gene of interest was calculated relative to the Gapdh mRNA (ΔC_T) and is presented as "fold induction" relative to values obtained with the respective control (set as "1-fold"). Primers are provided in S2 Table.
