# Supplementary figures and images for: Threshold Levels of Gfi1 Maintain E2A Activity for B Cell Commitment via Repression of Id1
Source: PLoS One. 2016 Jul 28;11(7):e0160344. doi: 10.1371/journal.pone.0160344 (PMC4965025; doi:10.1371/journal.pone.0160344)

**A**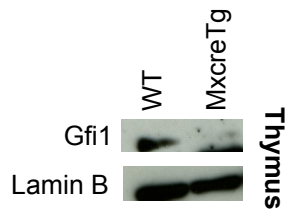**B**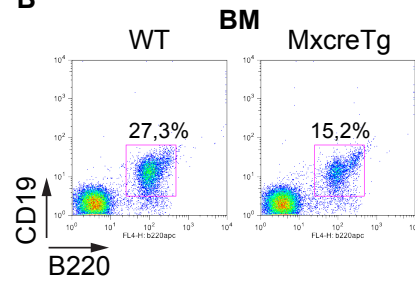**C**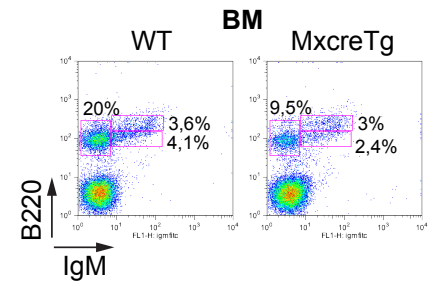**D**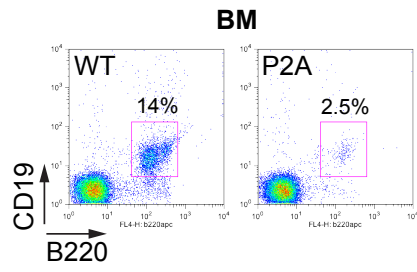**E**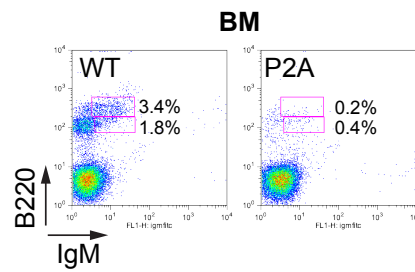**F**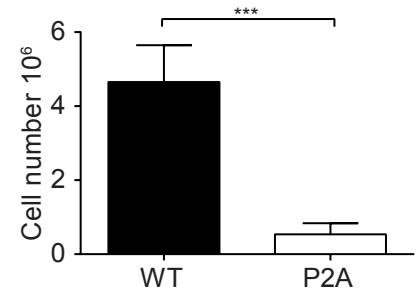

Supplement: S1 Fig — (A) Western blot Gfi1 from thymocytes isolated from Mx-cre Wt, Gfi1 flox/flox mouse (WT) treated with pIpC and Mx-cre Tg, Gfi1 flox/flox mice (MxcreTg) treated with pIpC to delete Gfi1. Lamin B was used as a loading control. (B) and (C) FACS analysis of bone marrow B cells from Mx-cre Wt, Gfi1 flox/flox mouse (WT) treated with pIpC and Mx-cre Tg, Gfi1 flox/flox mice (MxcreTg) treated with pIpC to delete Gfi1. Bone marrow cells were stained for B220 and CD19 (B) or B220 and IgM (C). (D) FACS analysis of B220 and CD19 in the bone marrow of WT and P2A mice. (E) FACS analysis of B220 and IgM in the bone marrow of WT and P2A mice. (F) Cell numbers of B220+ CD19+ cells in the WT and P2A bone marrow. At least three mice were used to determine absolute numbers of B cell subsets in bone marrow. (PDF) [file pone.0160344.s002.pdf]

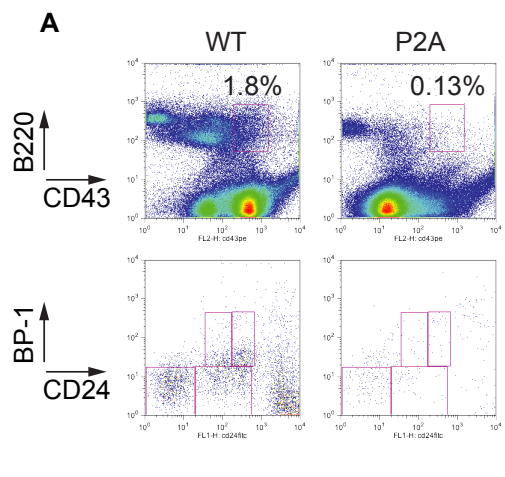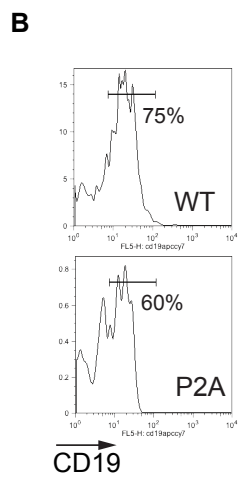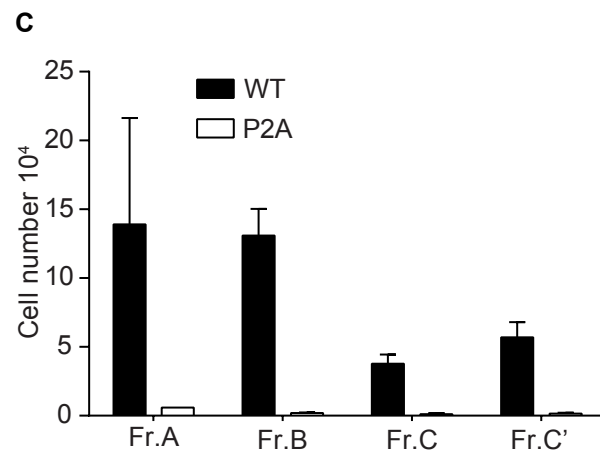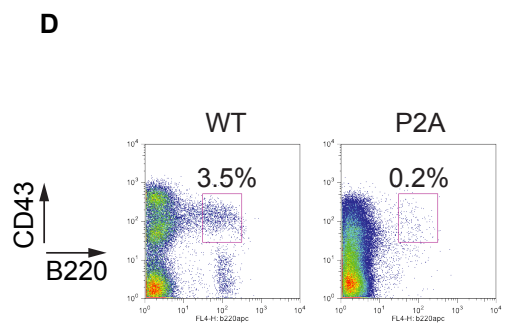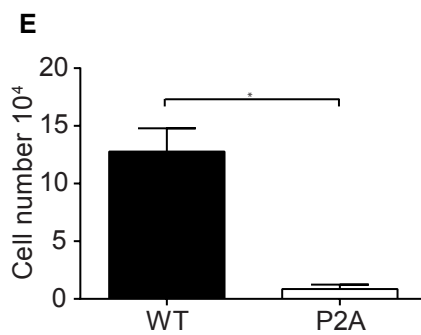

Supplement: S2 Fig — (A) Flow cytometry analysis of B220, CD43, HSA, and BP-1 surface expression on bone marrow cells from WT or P2A mice. B220+CD43+ cells were electronically gated and analyzed for HSA and BP-1 expression to identify Fraction A (Fr.A, B220+CD43+HSA-BP1−), Fraction B (Fr.B, B220+CD43+HSA+BP1−), Fraction C (Fr.C, B220+CD43+HSA+BP1+) and Fraction C’ (Fr.C’, B220+CD43+HSAhighBP1+). (B) CD19 expression on pro B cells from WT and P2A mice. (C) Absolute numbers of Fraction A-C’ from WT and P2A mice. (D) Expression of Lin-, CD19, B220, and CD43 on bone marrow cells from WT and P2A mice. Lin−CD19− cells were analyzed for the expression of B220 and CD43. (E) Absolute numbers of pre pro B cells in WT and P2A bone marrow. At least three mice were used to determine absolute numbers of B cell subsets in bone marrow. (PDF) [file pone.0160344.s003.pdf]

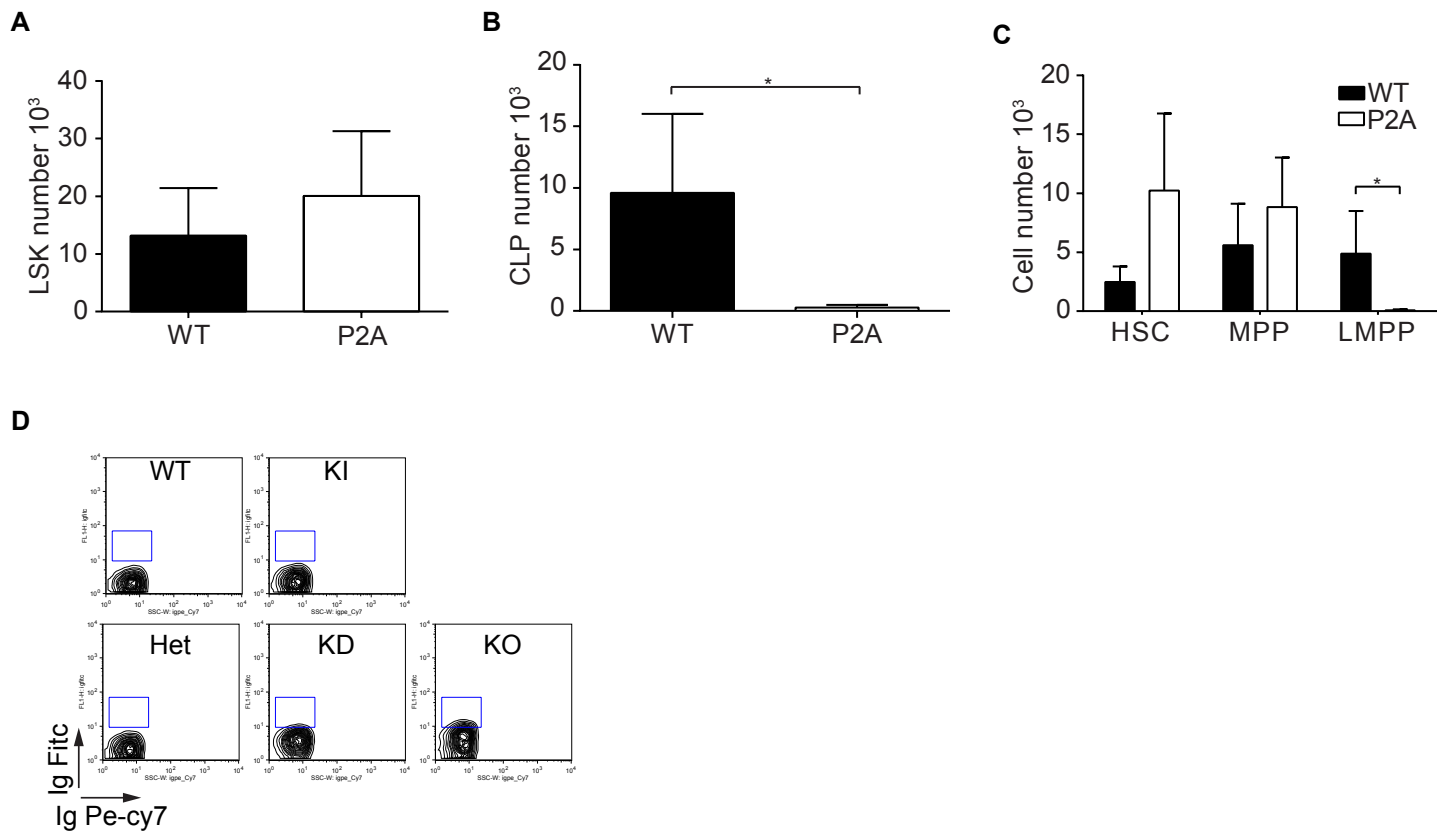

Supplement: S3 Fig — (A) LSK cell numbers in WT and P2A bone marrow. (B) CLP numbers in WT and P2A bone marrow. (C) Cell numbers of HSC, MPP and LMPP cells in the WT and P2A bone marrow. (D) BM cells from WT, KI, Het, KD and KO mice were stained with control isotypes to determine the CLP population gate. At least three mice were used to determine absolute numbers of B cell subsets in bone marrow. (PDF) [file pone.0160344.s004.pdf]

A

OP9 culture (B)

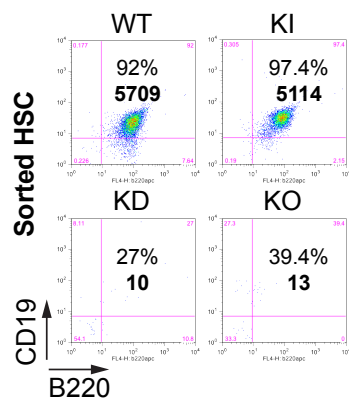

B

OP9 culture (B)

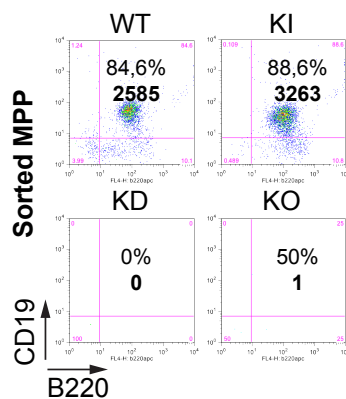

C

OP9 culture (B)

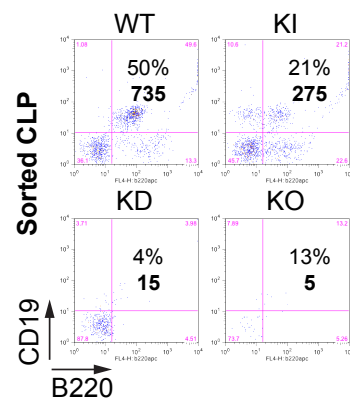

D

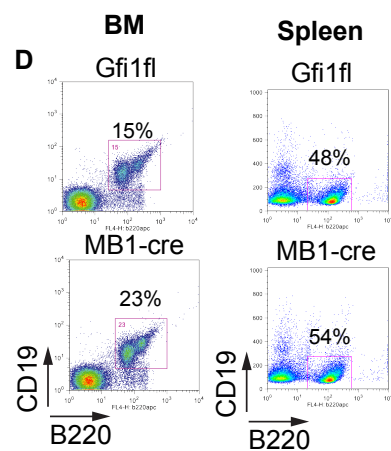

E

OP9-DL1 culture (T)

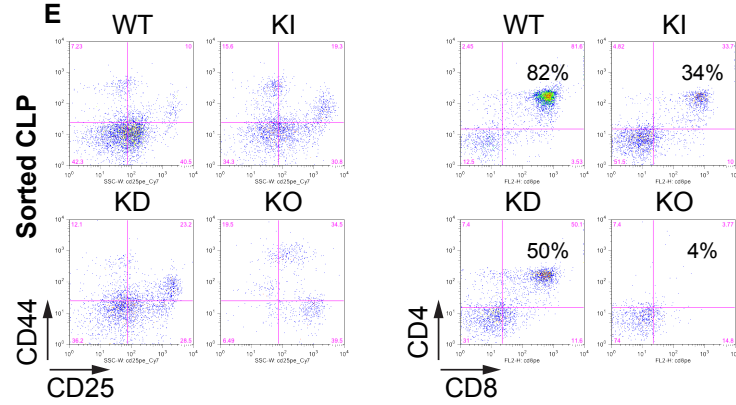

Supplement: S4 Fig — (A) HSC (Lin-Sca1+c-Kit+Flt3-), (B) MPP (Lin-Sca1+c-Kit+Flt3low) or (C) CLP (Lin-Il7R+Sca1+c-Kit+) were sorted and cultured on OP9 stromal cells in the presence of IL-7 (10 ng/mL) and Flt33L (5 ng/mL) for 10 or 12 days. Then cells were analyzed for B220 and CD19 expression by flow cytometry. The cell number on the FACS plots for each mouse is indicated in bold. (D) Gfi1 flox/flox (Gfi1fl), MB1-cre (MB1/WT) mice were analyzed for B cells in the bone marrow and spleen. (E) Sorted CLPs were cultured on OP9-DL1 cells in the presence of SCF (10 ng/mL), FLT3L (5 ng/mL) and IL-7 (1 ng/mL) for at least 15 days. The cells were analyzed for CD44, CD25, CD4 and CD8 expression. All FACS plots are representative of at least two independent experiments and at least three mice were used to determine absolute numbers of B cell subsets in bone marrow and spleen. (PDF) [file pone.0160344.s005.pdf]

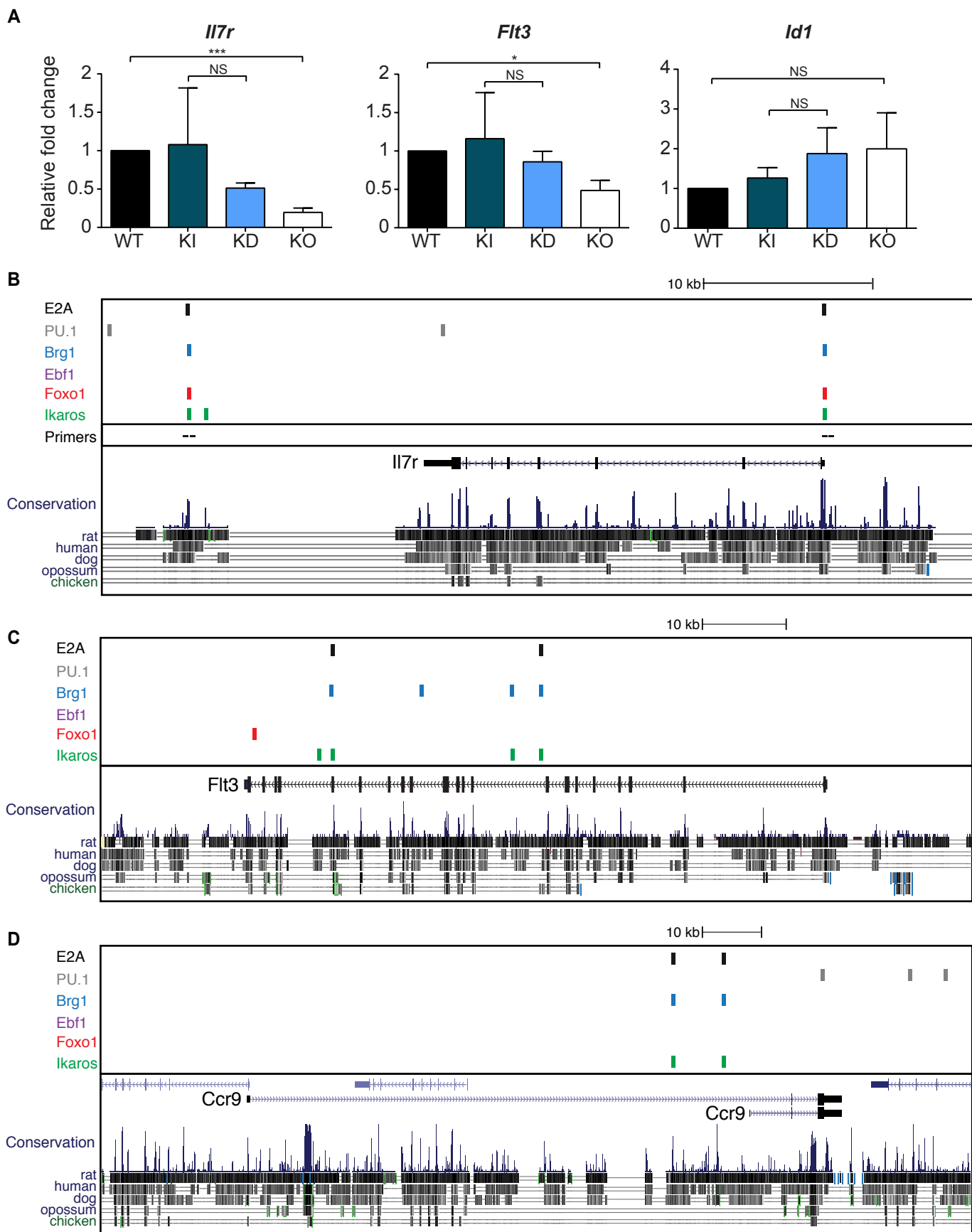

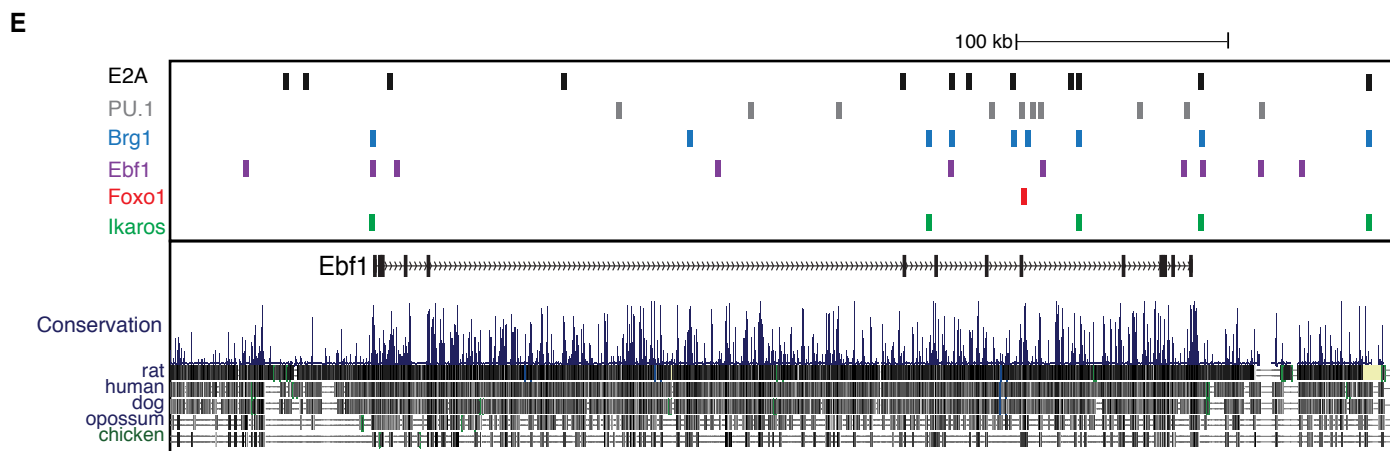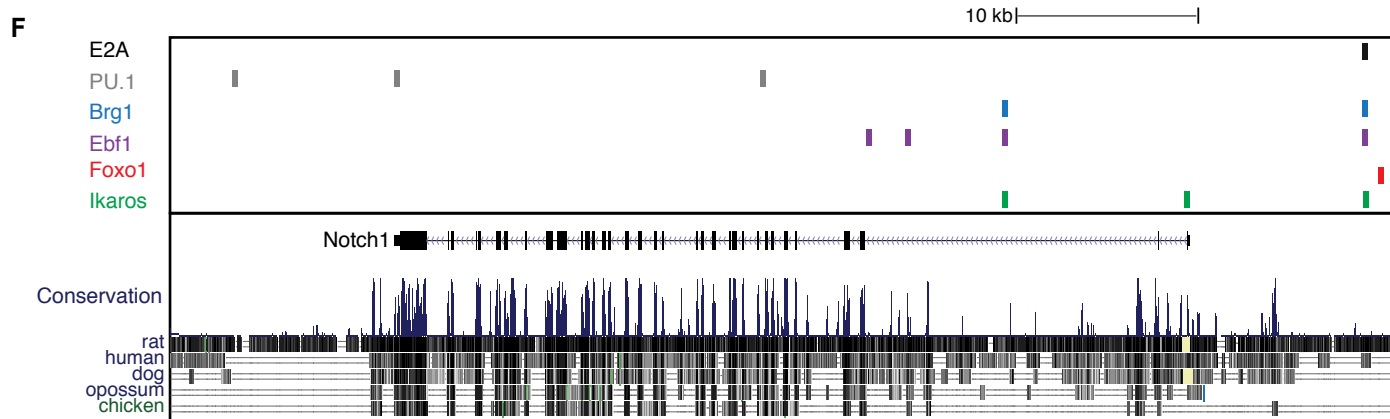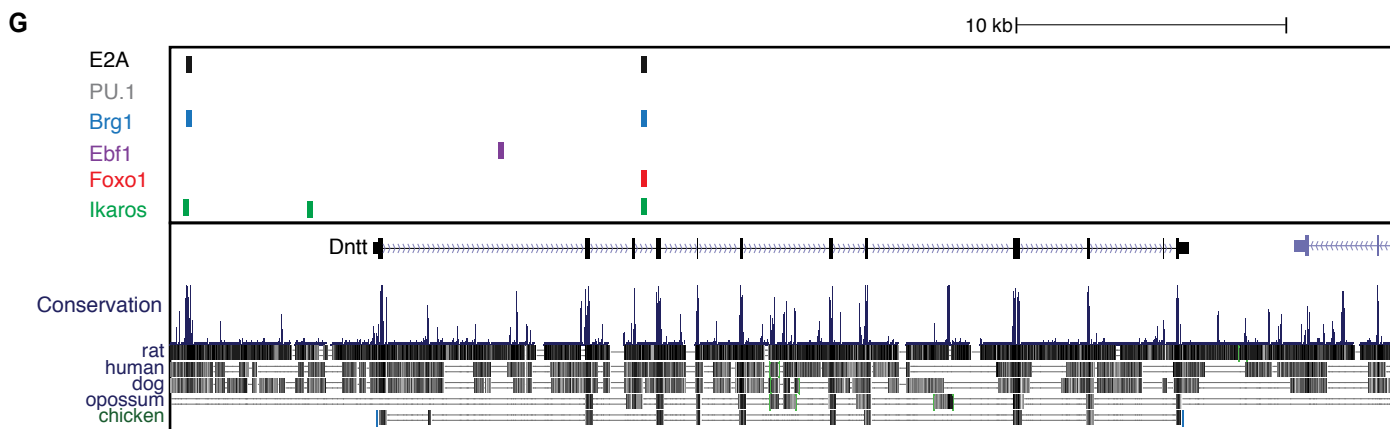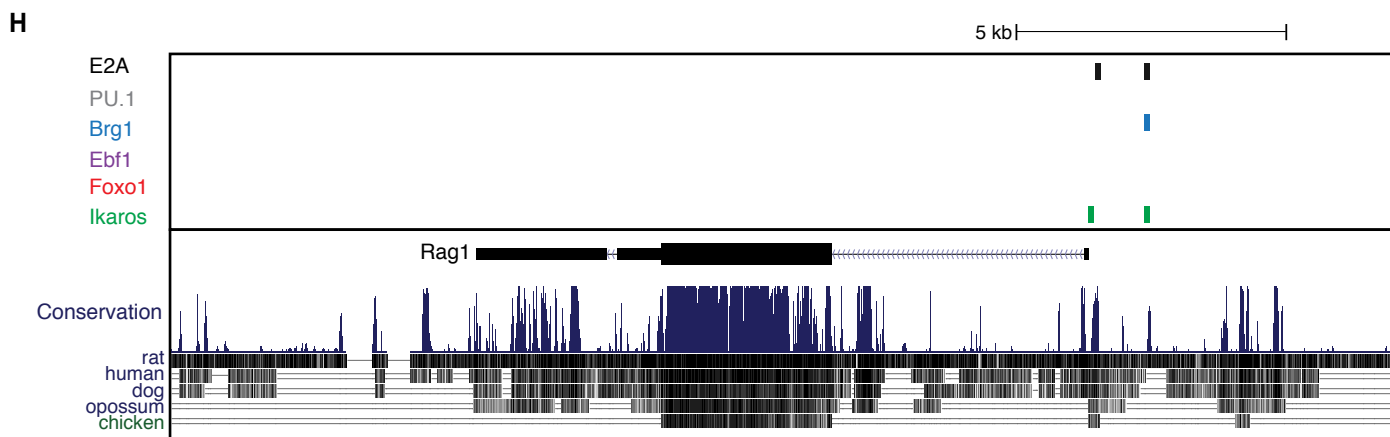

Supplement: S6 Fig — (A) Il7r, Flt3 and Id1 expression in sorted pre-pro B cells from WT, KI, KD and KO was measured by real time qPCR. Expression of Il7r, Flt3 and Id1 was normalized to Gapdh and presented as the fold increase relative to cDNA from WT cells. (B-H) E2A, PU.1, Brg1, EBF1, Foxo1 and Ikaros targeted regulatory regions from RAG1 null pro-B cells by ChIP-seq across the Il7r, (C) Flt3, (D) Ccr9, (E) Ebf1, (F) Notch1, (G) Dntt, (H) Rag1 loci. (PDF) [file pone.0160344.s007.pdf]
