# Supplementary material for: Threshold Levels of Gfi1 Maintain E2A Activity for B Cell Commitment via Repression of Id1
Source: PLoS One. 2016 Jul 28;11(7):e0160344. doi: 10.1371/journal.pone.0160344 (PMC4965025; doi:10.1371/journal.pone.0160344)

A

Gene Title	Symbol	Genbank ID	Log2 Fold Change (KO/WT)	P-value
interleukin 7 receptor	*Il7r*	AI573431	-1.63	8.2E-04
recombination activating gene 1	*Rag1*	NM_009019	-1.07	9.5E-02
FMS-like tyrosine kinase 3	*Flt3*	NM_010229	-1.00	3.4E-05
Deoxynucleotidyltransferase, terminal	*Dntt*	BB160593	-0.93	1.7E-03
notch gene homolog 1 (Drosophila)	*Notch1*	NM_008714	-0.81	3.1E-04
runt related transcription factor 1	*Runx1*	X97306	-0.14	3.3E-01
early B-cell factor 1	*Ebf1*	BB125261	-0.12	4.6E-01
GA repeat binding protein, alpha	*Gabpa*	NM_008065	-0.02	9.0E-01
chemokine (C-C motif) receptor 9	*Ccr9*	NM_009913	-0.01	9.5E-01
transcription factor 3	*Tcf3*	D29919	0.01	9.2E-01
Cd19 antigen	*Cd19*	NM_009844	0.06	7.4E-01
paired box gene 5	*Pax5*	NM_008782	0.07	6.5E-01
inhibitor of DNA binding 2	*Id2*	AK013239	0.15	2.2E-01
Immunoglobulin (CD79A) binding protein 1	*Igbp1*	C81413	0.18	3.4E-01
inhibitor of DNA binding 4	*Id4*	BB121406	0.25	9.5E-02
inhibitor of DNA binding 3	*Id3*	NM_008321	0.28	6.7E-02
colony stimulating factor 1 receptor	*Csf1r*	AI323359	0.89	2.3E-03
inhibitor of DNA binding 1	*Id1*	U43884	0.92	7.8E-06

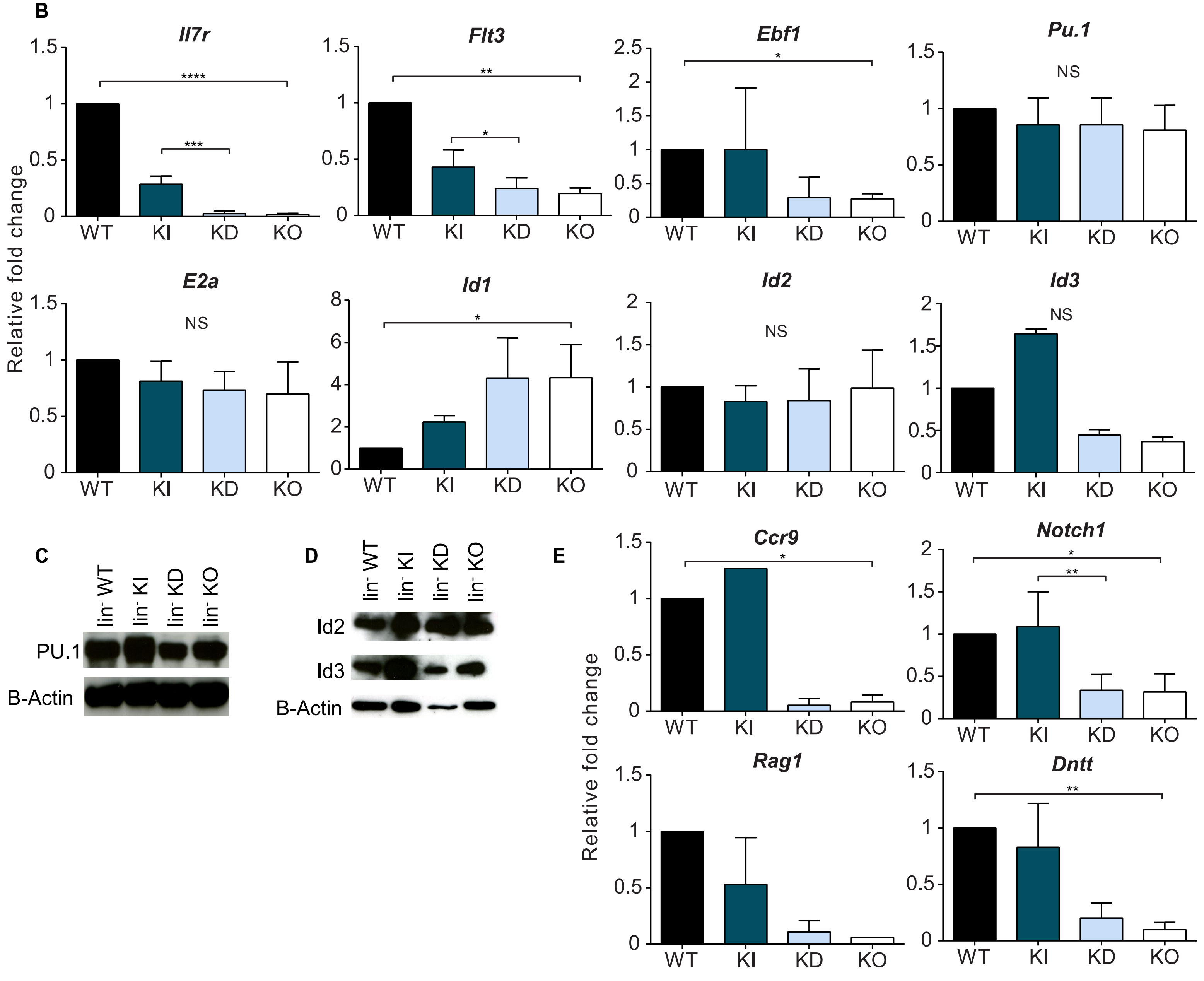

Supplement: S5 Fig — (A) Table showing the expression log2 fold change of selected up- and down-regulated genes between sorted WT and KO LSKs. (B) Il7r, Flt3, Ebf1, Pu.1, E2a, Id1, Id2 and Id3 expression in sorted LSK cells from WT, KI, KD and KO mice was measured by real time qPCR. Expression of the genes was normalized to Gapdh and is presented relative to cDNA from WT cells. (C) PU.1 expression in Lin- cells from WT, KI, KD and KO BM was measured by western blot. β-actin was used as a loading control. (D) Id2 and Id3 expression in lineage negative cells from WT, KI, KD and KO mice measured by western blot. β-actin was used as a loading control. (E) Ccr9, Notch1, Rag1 and Dntt expression in sorted LSK cells from WT, KI, KD and KO mice was measured by real time qPCR. Expression of the genes was normalized to Gapdh and is presented relative to cDNA from WT cells. Real time PCR are representative of at least 3 independent experiments and the western blot scans are representative of two different experiments. (PDF) [file pone.0160344.s006.pdf]
