# Supplementary material for: Threshold Levels of Gfi1 Maintain E2A Activity for B Cell Commitment via Repression of Id1
Source: PLoS One. 2016 Jul 28;11(7):e0160344. doi: 10.1371/journal.pone.0160344 (PMC4965025; doi:10.1371/journal.pone.0160344)
Supplement: S1 Table — (PDF) [file pone.0160344.s008.pdf]

S1 Table: Primers for mouse and human ChIP-qPCR

Mouse ChIP-qPCR primers	Sequences (5'-3')
Flt3 Promoter Forward	CGCGCATCACTCCCTCATAC
Flt3 Promoter Reverse	CAAAGCCAAGTTCACCGCTT
Gapdh Promoter Forward	ACTGAGCAAGAGAGGCCCTA
Gapdh Promoter Reverse	TATGGGGGTCTGGGATGGAA
Il7r Promoter Forward	CAGCAGCAATCCTTTTGCTCA
Il7r Promoter Reverse	TGCCTGCTAAACCACAGACA
Il7r Enhancer Forward	GCTGGGGAGGGAAATGTTGA
Il7r Enhancer Reverse	TGCAAGGAGTCAGTTGCCTT
Intergenic control Forward	TGGGCATATCCCTGGAGCTT
Intergenic control Reverse	GGCCATCCCACAGTCACAAC
Tnrc5 Promoter Forward	CCCGCTGCCTCTTATTTCCTTTG
Tnrc5 Promoter Reverse	ACCCAGTCGGTCTCCTCAG

Human ChIP-qPCR primers	Sequences (5'-3')
ID1 Forward	GTGGCCATCTCGCGCT
ID1 Reverse	AGCTCCTTGAGGCGTGAGTA
GAPDH Forward	CCACATCGCTCAGACACCAT
GAPDH Reverse	CCCGCAAGGCTCGTAGAC
Intergenic control Forward	CCTGGCCTCTCACACTCA
Intergenic control Reverse	AGAACCCTTGCTCTCCAC
