# Supplementary material for: Threshold Levels of Gfi1 Maintain E2A Activity for B Cell Commitment via Repression of Id1
Source: PLoS One. 2016 Jul 28;11(7):e0160344. doi: 10.1371/journal.pone.0160344 (PMC4965025; doi:10.1371/journal.pone.0160344)
Supplement: S2 Table — (PDF) [file pone.0160344.s009.pdf]

S2 Table: Primers for real Time PCR

Primers	Sequences (5’-3’)
Ccr9 Forward	CAATCTGGGATGAGCCTAAACAAC
Ccr9 Reverse	ACCAAAAACCAACTGCTGCG
Dntt Forward	TCATGTGCCCCTATGATCGC
Dntt Reverse	ATCGTGACAGTCTTCCCCTT
E2a Forward	CATGCCCCCGCCCAG
E2a Reverse	CAGTTCCTTGTCAGAGCCCA
Ebf1 Forward	TGCCATCCGAGTTCAGACAC
Ebf1 Reverse	ATCTGCCTGGTGTCCCTTTG
Flt3 Forward	AGTCTCAATTCAGGTGGCGG
Flt3 Reverse	CCCCAGCAGATTCACGATGT
Id1 Forward	TGGGAAAGACACTACCGCAG
Id1 Reverse	CTCTGGAGGCTGAAAGGTGG
Id2 Forward	ACTCGCATCCCACTATCGTC
Id2 Reverse	GATGTCCGTGTTCAGGGTGG
Id3 Forward	CTCTCCAACATGAAGGCGCT
Id3 Reverse	CGTCCAAGAGGCTAAGAGGC
Il7r Forward	AGCAAGGGGTGAAAGCAACT
Il7r Reverse	AACTTGGACTCCACTCGCTC
Notch1 Forward	CCTCCTGCCAGAACACCAAT
Notch1 Reverse	TCACTCTCACAGTTGCGACC
Pax5 Forward	AGTGTCTACAGGCTCCGTGA
Pax5 Reverse	CCCTCTTGCGTTTGTTGGTG
Pu.1 Forward	CAGCAGCTCTATCGCCACAT
Pu.1 Reverse	ATCCGGGGCATGTAGGAAAC
Rag1 Forward	TTAACAACCAAGCTGCAGACA
Rag1 Reverse	AGCTCAGGGTAGACGGCAA
